# Identification and comprehensive analysis of an immune-related gene prognostic model for indicating tumor immune microenvironment features in soft tissue sarcoma

**DOI:** 10.3389/fonc.2025.1609501

**Published:** 2025-09-03

**Authors:** Shizhao Xu, Yuqiang Liu, Qian Cai, Jianzhi Sun, Xuefeng Guan

**Affiliations:** ^1^ The Ministry of National Education Key Lab for Traditional Chinese Medicine Visceral Manifestations Theory and Application, Liaoning University of Traditional Chinese Medicine, Shenyang, China; ^2^ Department of Clinical Pharmacy, Dalian Municipal Central Hospital Affiliated of Dalian University of Technology, Dalian, China

**Keywords:** soft tissue sarcoma, immune-related genes, tumor microenvironment, immune cell infiltration, prognostic model signatures

## Abstract

**Background:**

the STS is a rare type of tumor, and although its treatment has improved greatly in recent decades, the treatment of STS and the development of new drugs remains a major challenge. A new identification of prognostic biomarkers that reflect the biological heterogeneity of STS could therefore lead to better interventions for STS patients. In recent years, there has been a growing interest in the investigation of the impact of immune-related genes on cancer prognosis.

**Methods:**

based on RNA-seq data obtained from TCGA-STS and GTEx patients, differential expression analysis, consensus clustering, enrichment analysis, tumor microenvironment assessment, risk model construction and other data analysis were performed. Last but not least, CALR, a central regulator inSTS, demonstrated oncogenic properties through overexpression/knockdown assays, supported by qRT-PCR and immunofluorescence data.

**Results:**

we constructed a prognostic model containing 8 IRGs for predicting STS prognosis by using the LASSO regression. Furthermore, the samples were categorized as either high-risk or low-risk based on the risk score computed by the model. Additionally, we compared the tumor microenvironment of STS samples using the ESTIMATE and CIBERSORT algorithms. Last, our experimental results proved that CALR was up-regulated in sarcoma cells compared to in normal cell.

**Conclusions:**

conclusively, IRGPM is a promising immune-related prognostic biomarker. As a prognostic indicator of immunotherapy, IRGPM might also help differentiate molecular and immune characteristics in STS.

## 1 Introduction

Soft Tissue Sarcoma (STS) refers to a collection of cancerous tumors that arise from non-epithelial extraosseous tissues. The incidence of STS is low, about 29100 in China, and 40% to 60% of patients develop hematogenous metastasis during the disease progression (1). The diagnosis and treatment of STS requires multidisciplinary cooperation. Although chemotherapy is currently the most widely used systemic therapy, the indications and protocols of chemotherapy for STS have not been as clear and uniform as those for other tumors for a long time, mainly because of the low incidence of STS, the variety of tissue types, and the different biological behaviors, which have very different sensitivity to chemotherapy. Therefore, to improve prognosis, STS biomarkers associated with biological heterogeneity need to be identified (2, 3). Currently, immune oncology is attracting much attention due to its particular benefits for cancer patients. Immunotherapy, a new therapeutic approach, has demonstrated encouraging outcomes in combating specific types of cancer, including breast cancer and hepatocellular carcinoma (4, 5). The involvement of immune-related genes and immune cells is essential in the development and progression of tumors (6, 7). As a result, this comprehensive study may provide insight into new treatment and prognostic factors for STS based on the relationship between immune-related genes. An 8-immune related gene prognostic model (IRGPM) was identified to be significantly correlated with prognosis in both groups of Soft Tissue Sarcoma by utilizing univariate Cox and LASSO regression analysis. Additionally, we compared the tumor microenvironment of STS samples using the ESTIMATE and CIBERSORT algorithms. The immune-related prognostic biomarker, IRGPM, has been recognized as a promising candidate for predicting the prognosis of STS. As a prognostic indicator of immunotherapy, IRGPM might also help differentiate molecular and immune characteristics in STS.

## 2 Materials and methods

### 2.1 Patients and datasets

**Figure 1:**
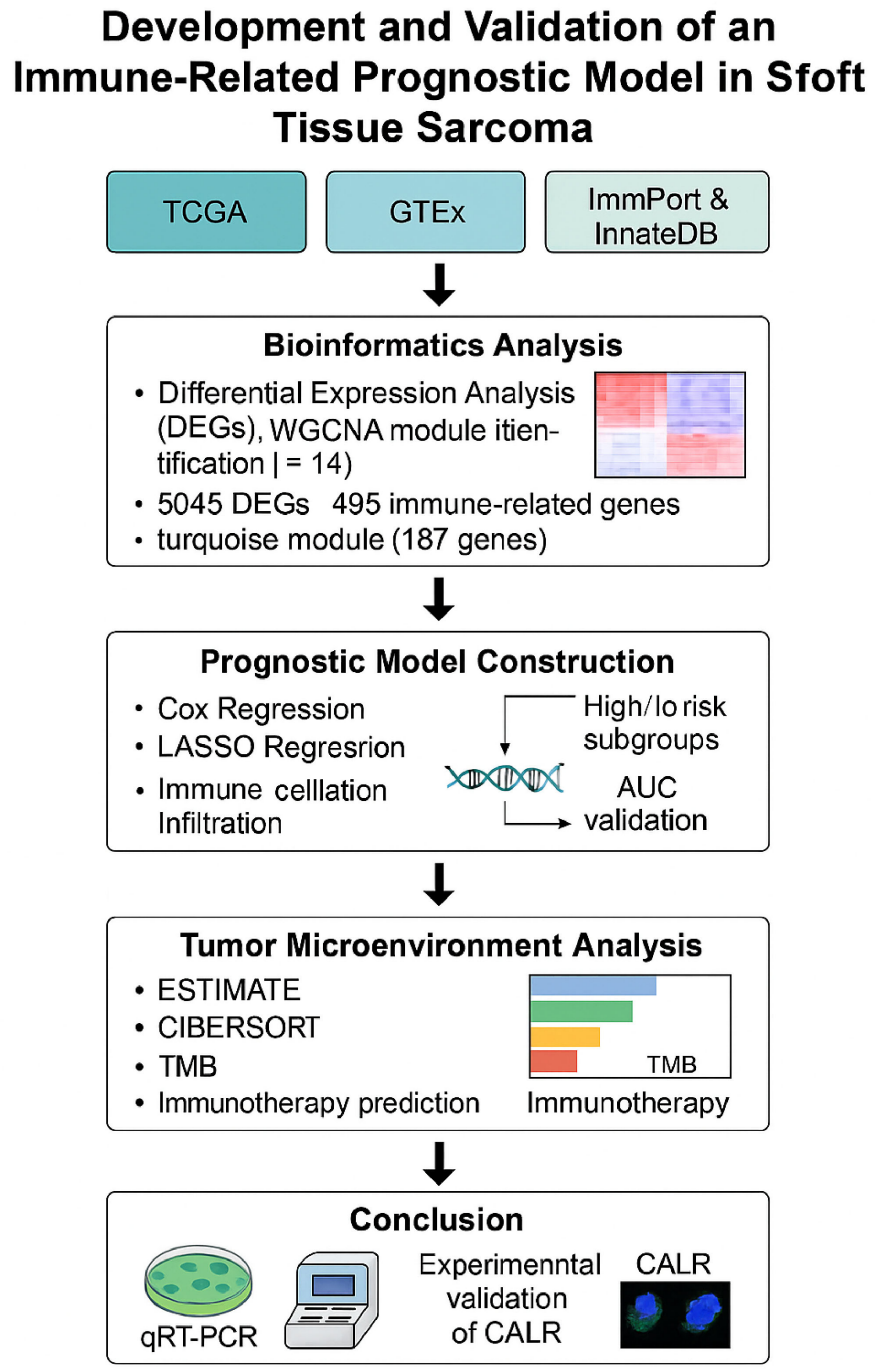
Workflow of immune-related prognostic modeling and validation in soft tissue sarcoma.

As shown in **Figure 1**, The Cancer Genome Atlas (TCGA)-STS and The Genotype-Tissue Expression (GTEx) database (https://xenabrowser.net/datapages/) were used to download RNA sequencing data from 392 samples, including 263 STS samples, 129 normal samples as well as their clinicopathologic characteristics. The ImmPort (https://www.immport.org/shared/home) and InnateDB (https://www.innatedb.com/) databases were used to download immune-related gene lists.

### 2.2 Identification of immune-related hub genes

On the basis of RNA-seq data obtained from TCGA-STS and GTEx patients (263 tumors vs 129 normal samples), Using the package limma, differentially expressed genes (DEGs) (P<0.05, |log2FC| >1) were identified. Using the immune-related gene (IRG) lists from ImmPort and InnateDB, and the clusterProfiler package of R, we acquired and analyzed immune-related differentially expressed genes based on Gene Ontology (GO) and Kyoto Encyclopedia of Genes and Genomes (KEGG) analyses.

WGCNA was then used to identify hub genes. In the first step, A correlation coefficient derived from the expression data of a pair of IRGs was used to calculate the similarity matrix. The similarity matrix was first converted into an adjacency matrix utilizing a signed network type and β=14. To construct a scale-free co-expression network, we calculated the soft-thresholding power (β) using the pickSoftThreshold function in the WGCNA R package. The value of β = 14 was chosen based on the criterion that the scale-free topology fit index (R²) exceeds 0.85, while maintaining mean connectivity at a reasonable level. This ensures the resulting network conforms to a scale-free distribution, which is biologically meaningful for gene co-expression analysis (see **Supplementary Figure X for the scale-free topology plot**). Subsequently, the topological overlap measure (TOM) was used to transform the adjacency matrix into a topological matrix, which described the level of gene association. A dynamic pruning tree was constructed using the 1-TOM distance metric between genes to identify modules. Last but not least, in order to identify four modules, the merge threshold function was set to 0.25. The genes located in the turquoise module were identified as the hub genes. In order to construct the network, genes with weight > 0.2 from significant related modules (turquoise module) were connected. To establish the optimal cut-off value for each hub gene with respect to overall survival (OS), the survminer package in R was employed. Forty-six hub genes associated with immune response and exhibiting significant survival association (log-rank test, P < 0.05) were selected for further analysis. The maftools package was utilized to identify relevant mutations in the analysis of the 46 immune-related hub genes.

### 2.3 Construction and validation of the IRGPM Among 187 immune-related hub genes

In our study, 256 patients with STS were randomly assigned to one of two groups (2:1). In the training group, a prognostic model for immune related genes was developed by combining univariate Cox regression, LASSO Cox regression, and multivariate Cox regression. As a result of using this prognostic model, an individual’s risk score was calculated. In accordance with the formula below, a risk score was calculated: risk score = the normalized expression level and corresponding correlation coefficient of each immune related gene were multiplied. The median risk score was used to categorize the training, testing, and all patient groups as either high risk or low risk. The high risk and low risk groups were compared in terms of OS in the training group, testing group, and all patients, as determined by Kaplan Meier analysis.

### 2.4 Relationship between IRGPM grouping and other molecular, immune subtypes

We identified the gene sets enriched in each IRGPM subgroup through GSEA (GO, KEGG), and proceeded to gain additional biological insight by analyzing gene mutations using R’s maftools package. The distribution of immune subtypes within the IRGPM groups was then examined. In this study, the component of C1, C2, C3, C4, and C6 between IRGPM-high subgroup and IRGPM-low subgroup were compared. (P < 0.001). As part of our evaluation of STS’s immune microenvironment, using the CIBERSORT algorithm, we calculated the proportion of immune cells that had infiltrated the tumor in every patient (8) A 0.05 P value was applied to the results produced by CIBERSORT. In addition, clinicopathologic factors and immune cell proportions between the two IRGPM subgroups were compared. A correlation analysis was conducted on 256 STS samples to determine the immune characteristics by examining the connection between the IRGPM score and the total mutation burden (TMB). Calculation of the TIDE score was done using a web-based calculator (http://tide.dfci.harvard.edu/).

### 2.5 Cell culture and qRT-PCR analysis

Sarcoma cell lines (143B, MG63, U2OS, SaOS, HOS) and human osteoblasts (hFOB1.19) were obtained from ATCC (Manassas, VA, USA). Cells were cultured in DMEM medium (Solarbio, Beijing, China) supplemented with 10% fetal bovine serum (FBS) and 1% penicillin-streptomycin, and maintained at 37°C with 5% CO_2_. Total RNA was extracted using TRIzol reagent (Invitrogen, Carlsbad, CA, USA), and cDNA was synthesized using the ReverTra Ace qPCR RT Kit with gDNA Remover. Quantitative real-time PCR (qRT-PCR) was performed using SYBR Premix Ex Taq II on an Mx3005P real-time PCR system (Stratagene, San Diego, CA, USA), with GAPDH as an endogenous control. qRT-PCR conditions included 95°C for 10 min (pre-denaturation), 95°C for 5 s (denaturation), 60°C for 30 s (annealing), and 45 cycles. Each sample was analyzed in triplicate, and gene expression levels were calculated using the 2^(-ΔΔCt) method, with primer sequences provided in **Supplementary Table 1**.

### 2.6 Overexpression plasmid, shRNA and antibodies

CALR shRNA (#sc-141687-SH) and CALR overexpression plasmid (#EX-Mm07902-M61) were purchased from Santa Cruz Biotechnology and GeneCopoeia, respectively. Transfection was performed using Lipofectamine 3000 reagent (#L3000008, Thermo Fisher Scientific). Anti-CALR (#10208-1-AP, 1:1000) and anti-GAPDH (#ab8245, 1:2000) antibodies were purchased from Proteintech and Abcam, respectively. Anti-Flag antibody (#F3165, 1:5000, Sigma-Aldrich) was used to detect overexpression constructs (**Supplementary Tables 2**, **3**).

### 2.7 Western blot, and immunofluorescence staining

Total proteins from sarcoma cell lines (143B, MG63, U2OS, SaOS) were lysed using RIPA buffer, separated by 10% SDS-PAGE, and transferred to PVDF membranes. Following blocking with 5% skim milk, the membranes were incubated with primary antibodies overnight at 4°C, then probed with secondary antibodies for 2 hours at room temperature prior to centrifugation (4°C). Protein bands were quantified using ImageJ, with statistical analysis performed in GraphPad Prism 9. Image contrast adjustments were applied uniformly in Photoshop.

143B cells were divided into three groups: CALR overexpression (OE), CALR knockdown, and negative control (NC). Cells were transfected with CALR overexpression plasmid (EX-H0146-M61, GeneCopoeia), CALR shRNA (sc-141687-SH, Santa Cruz Biotechnology), or scrambled shRNA (sc-108060, Santa Cruz Biotechnology) using Lipofectamine 3000 (#L3000008, Thermo Fisher) for 48 hours. Cells on coverslips were fixed with 4% PFA, permeabilized with 0.2% Triton X-100, blocked with 3% BSA, and incubated with anti-CALR antibody (#10208-1-AP, 1:200, Proteintech) overnight at 4°C, followed by TRITC-conjugated secondary antibody (1:500) and DAPI (1 μg/mL) at RT. Images were captured via Zeiss LSM 880 confocal microscope and analyzed with ImageJ.

### 2.8 Statistical analysis

All statistical analyses were performed using R (v4.0.5). To compare the distributions of transformed categorical variables, either Chi square or Mann Whitney U tests were conducted. A significance level of p<0.05 was assumed unless otherwise specified.

## 3 Results

### 3.1 Identification of immune-related hub genes

**Figure 2:**
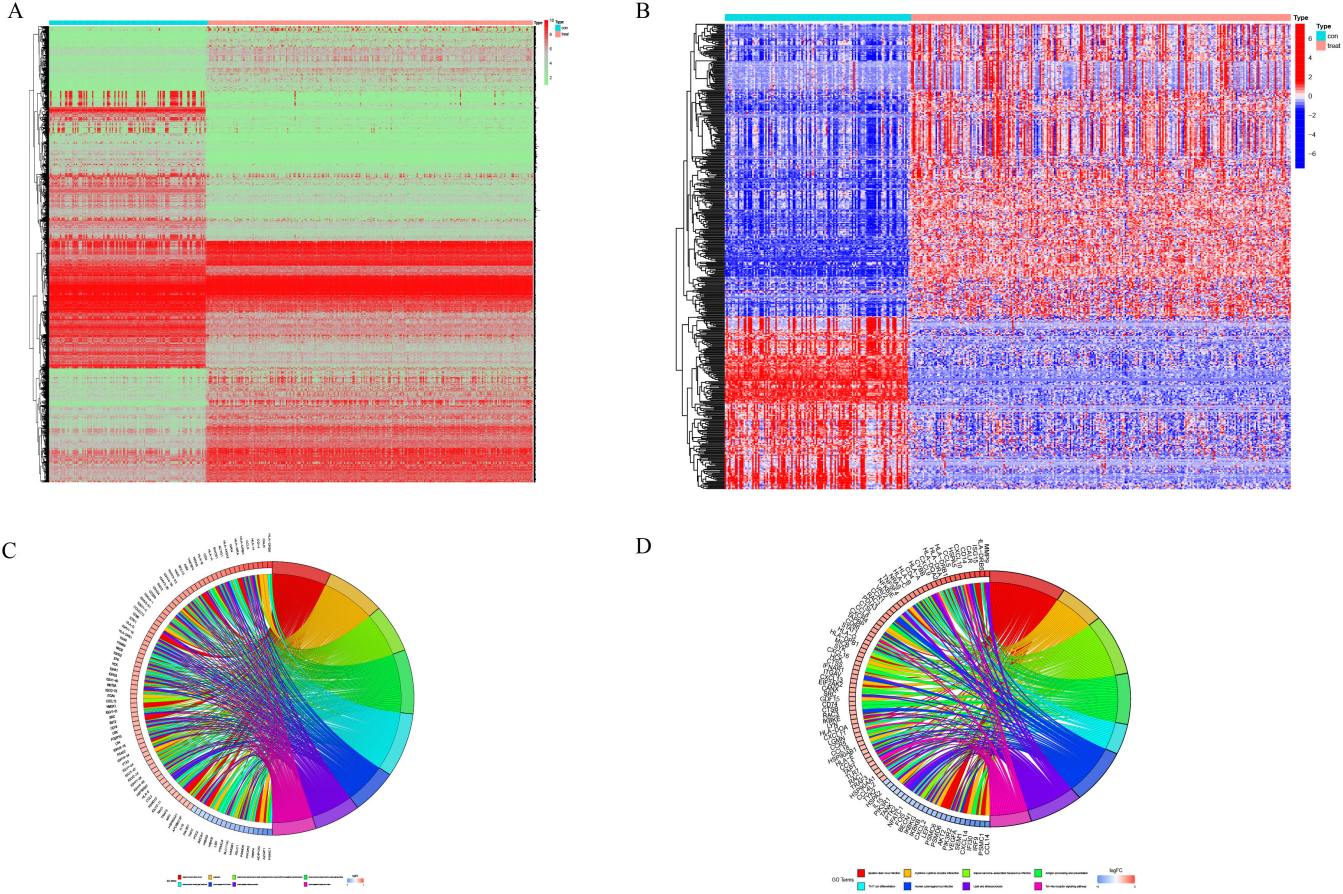
Differentially expressed immune‐related genes in STS. **(A)** A heatmap is presented to show the DEGs between 263 STS samples and 129 normal samples. **(B)** STS exhibited differential expression of immune related genes. **(C)** Enrichment analysis of immune related DEGs using Gene Ontology (GO). **(D)** Enrichment analysis of immune related DEGs using Kyoto Encyclopedia of Genes and Genomes (KEGG) pathway.

**Figure 3:**
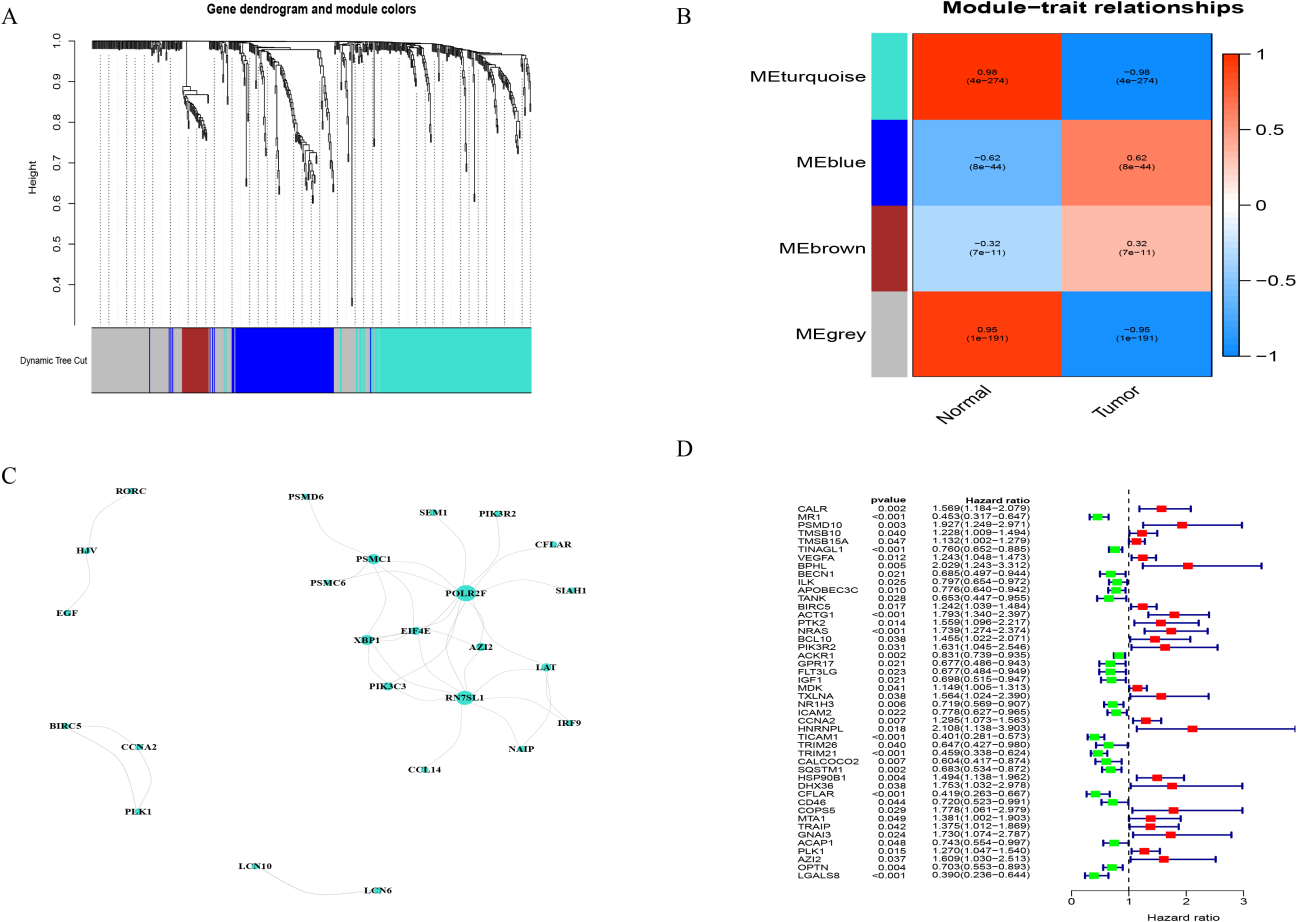
Identification of immune-related hub genes. **(A)** A soft threshold β = 5 was used for the Weighted Gene Co Expression Network Analysis (WGCNA) of immune related genes that showed differential expression. **(B)** The turquoise module is comprised of a network of genes. **(C)** WGCNA was used to obtain gene modules that are associated with overall survival. **(D)** Shown in the forest plots is the univariate Cox regression analysis between IRGPM and OS of STS. The P-values were obtained by univariate cox regression.

**Figure 4:**
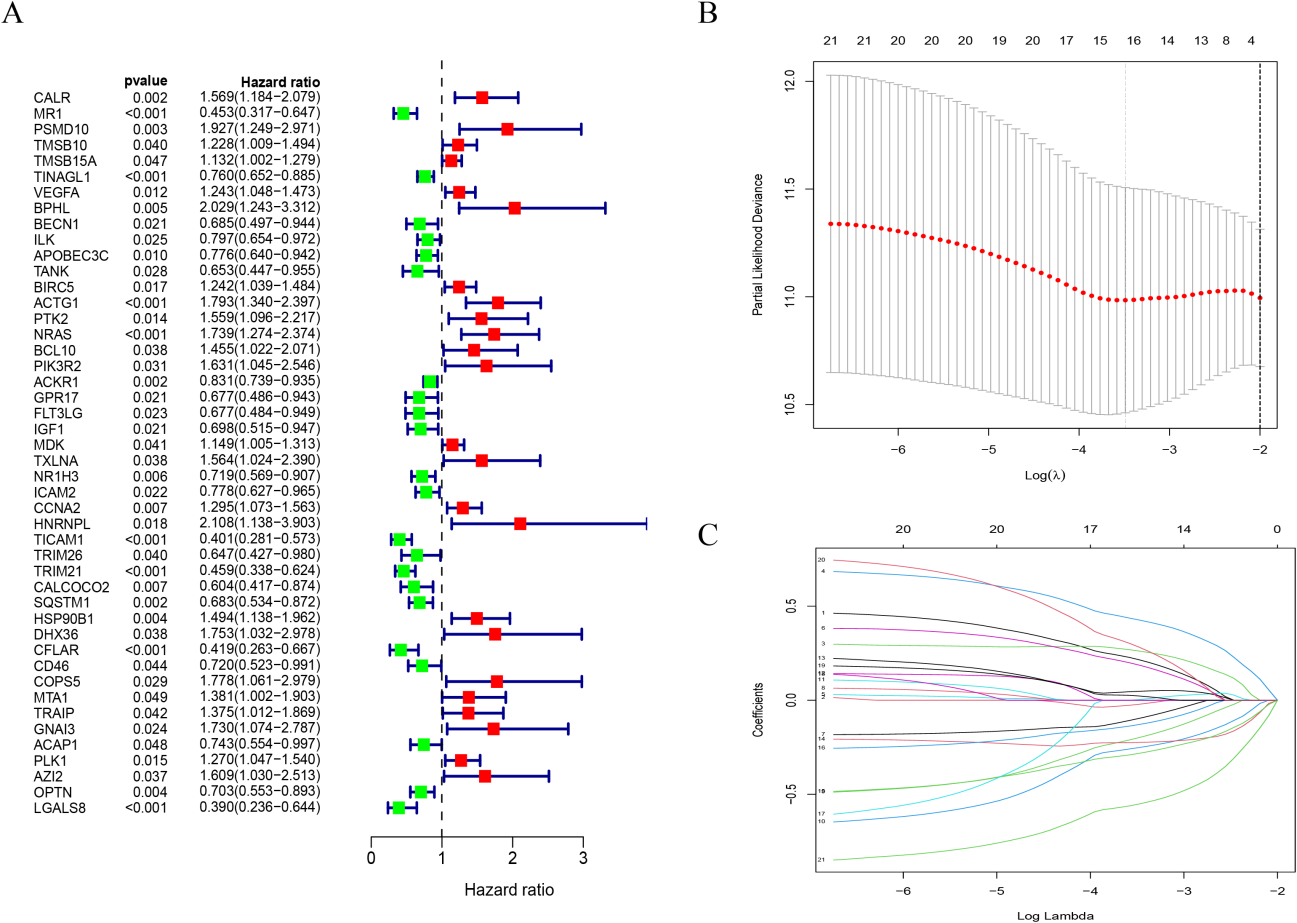
The construction of a prognostic model in OS patients. **(A)** There are changes in mutations and expression for 46 DEIRGs in patients. **(B)** By means of the LASSO regression model, only 8 genes related to immunity were chosen, meeting the minimum standards. **(C)** The immune related gene coefficients were calculated in LASSO regression.

In tumor samples compared to normal samples, there were a total of 5045 genes showing differential expression, with 2406 genes upregulated and 2639 genes downregulated (**Figure 2A**). Together with the immune-related genes lists obtained from ImmPort and InnateDB databases, a total of 495 genes with differential expression in immune cells have been identified. Among these genes, 311 showed upregulated expression in tumor samples, while 184 genes showed downregulated expression (**Figure 2B**). According to a functional enrichment analysis, 495 differentially expressed genes showed significant associations with KEGG pathways and GO terms. GO enriched in items such as regulation of immune effector process, receptor ligand activity and external side of plasma membrane (**Figure 2C**). KEGG enriched in pathways such as Cytokine−cytokine receptor interaction, MAPK signaling pathway, PI3K−Akt signaling pathway (**Figure 2D**). A further analysis of 495 differentially expressed genes was conducted by WGCNA. The correlation coefficient of probability of a node was greater than 0.9 between logarithm log(k) and logarithm log(P(k)), with the latter correlated negatively with logarithm log(k). We determined a soft threshold power of 14 by relying on the scale free network. Four modules were identified by utilizing the average linkage hierarchical clustering and the optimal soft threshold power (**Figure 3A**). Across 4 modules, 495 genes are present. Based on the module’s Pierson correlation coefficient, it is found that turquoise module (187 genes) was closely associated with STS tumors (**Figure 3C**). We conducted a correlation analysis on the 187 hub immune related genes in this module. A total of 25 genes and 35 edges were identified in networks with a threshold weight > 0.2 within the turquoise module (**Figure 3B**). Based on Cox regression analysis and K–M analysis, OS showed a strong correlation with 46 hub genes (P = 0.05, logrank test) **Figure 3D**, **Supplementary Figure 1**. Following that, we examined the somatic mutation characteristics of 46 hub genes. A few of the 46 hub genes related to immune function were found to have deletion and missense mutations, with PTK2, TICAM1, and TRIM26 having mutation rates exceeding 1%. This was a notable discovery. (**Figure 4A**).

### 3.2 Construction and validation of the IRGPM prognostic model

**Figure 5:**
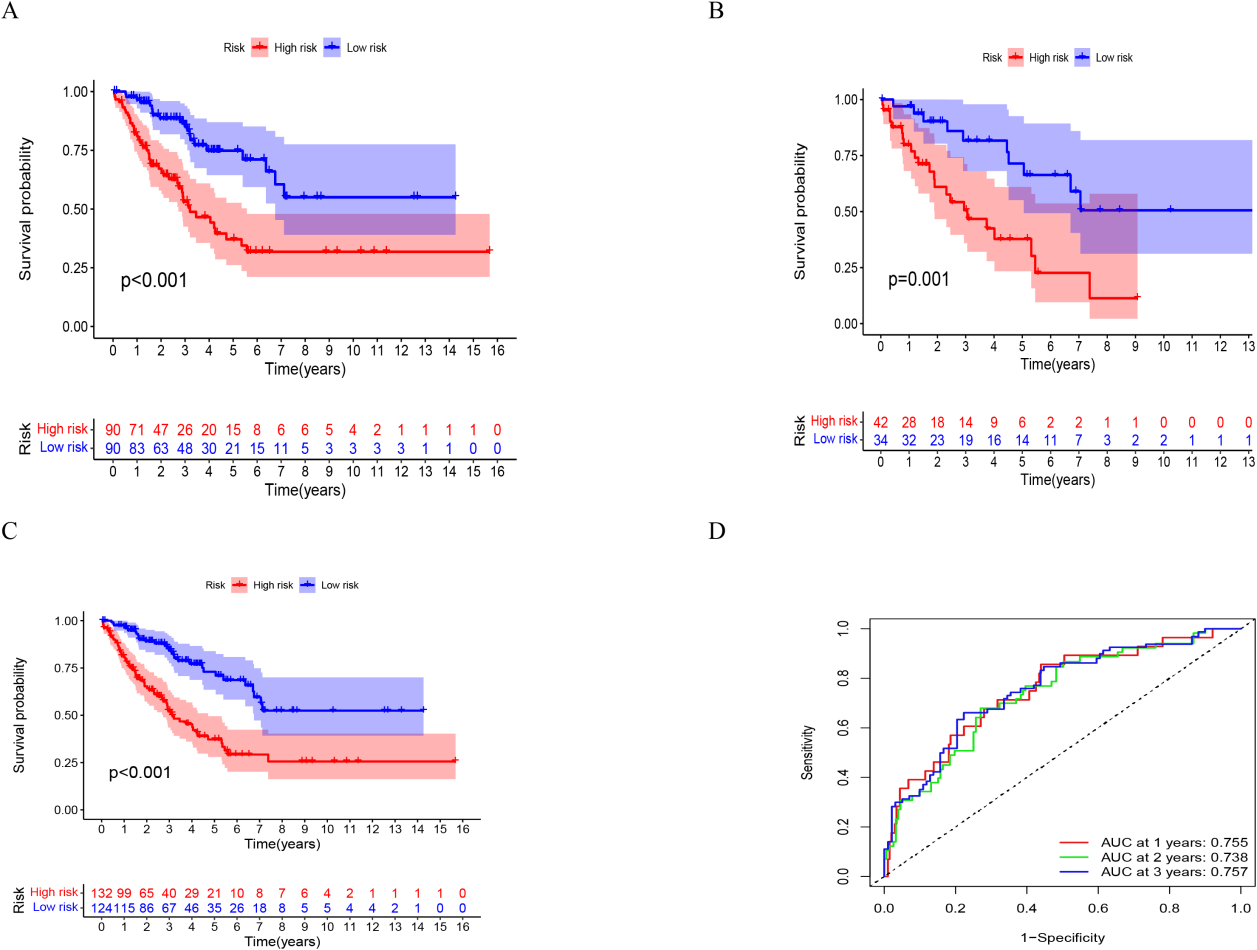
The validation of a prognostic signature in OS patients. **(A-C)** KM curves illustrate the overall survival in the training group, testing group, and all patients categorized into high and low risk groups. **(D)** The ROC curve verified the prognostic accuracy of the risk scores in all patients. ROC, receiver operating characteristic.

An average ratio of 2:1 was used for dividing 256 STS patients into two groups: the training group (n = 180) and the testing group (n = 76). The 187 hub genes in our training group were narrowed by LASSO Cox regression, resulting in 16 hub genes being chosen (**Figure 4B**). Following a subsequent multivariate analysis, the 8 IRGs with the lowest AIC (CALR, PSMD, IGF1, IL10RB, TRIM21, SQSTM1, AZI2, LGALS8) were identified as being prognostic (**Figure 4C**). In this section, we describe how risk scores are computed based on gene expression levels = CALR×0.43866885898529, PSMD10×0.658917776760825, IGF1×-0.382053790602671, IL10RB×-0.45324723287495, TRIM21×-0.4532805497586, SQSTM1×-0.26057468186444, AZI2×0.53318212745604, LGALS8×-0.770285176804154. Based on median risk scores, we split the training group into two groups one with high risk (n = 90) and another with low risk (n = 90). The Kaplan Meier analysis revealed that the high risk group had a considerably poorer OS compared to the low risk group. (**Figure 5A**). We used the same formula to calculate risk scores in the testing group as we did in the training group to verify the accuracy of the immune related gene prognostic model. Using the identical cut off values as those employed for the training group, the subjects were separated into two categories: the high risk group (n = 42) and the low risk group (n = 34). A relatively poor prognosis was observed in STS patients with high risk, according to Kaplan Meier analysis (**Figure 5B**). Using the identical formula applied to both the training and testing groups, a risk score was determined for each patient. Subsequently, patients were classified into two groups the high risk group (consisting of 132 patients) and the low risk group (consisting of 124 patients). Similarly, a relatively poor prognosis was observed in STS patients with high risk according to Kaplan Meier analysis. (**Figure 5C**). The AUC was 0.755 at 1 year, 0.738 at 2 years, and 0.757 at 3 years (**Figure 5D**).

### 3.3 Relationship between IRGPM grouping and other molecular, immune subtypes

**Figure 6:**
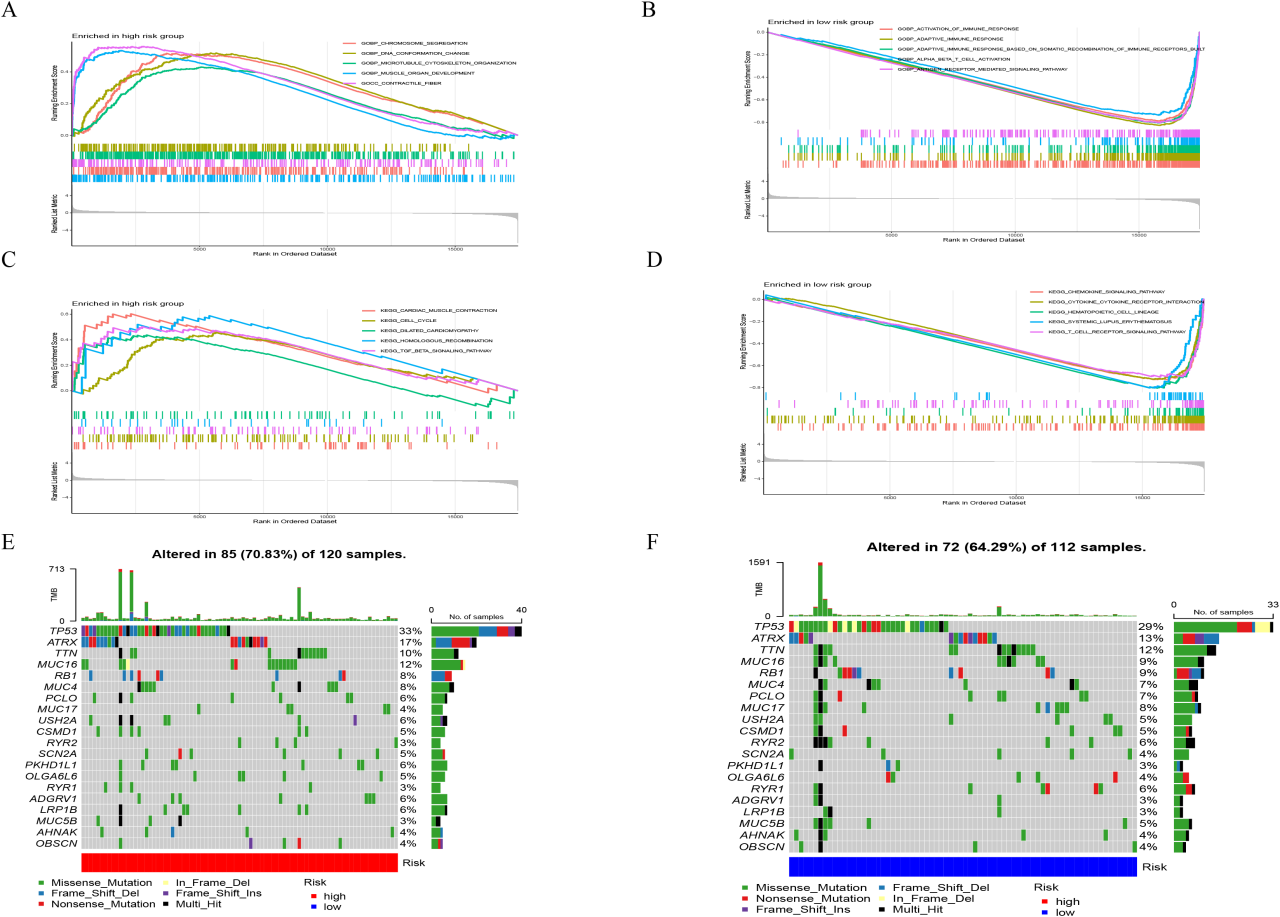
Molecular characteristics of different IRGPI subgroups. **(A)** Gene sets (GO) enriched in IRGPM-high subgroup. **(B)** Gene sets (GO) enriched in IRGPM-low subgroup. **(C)** Gene sets (KEGG) enriched in IRGPM-high subgroup. **(D)** Gene sets (KEGG) enriched in IRGPM-low subgroup. **(E, F)** Mutation changes and expression changes of DEIRGs in different IRGPM subgroups.

**Figure 7:**
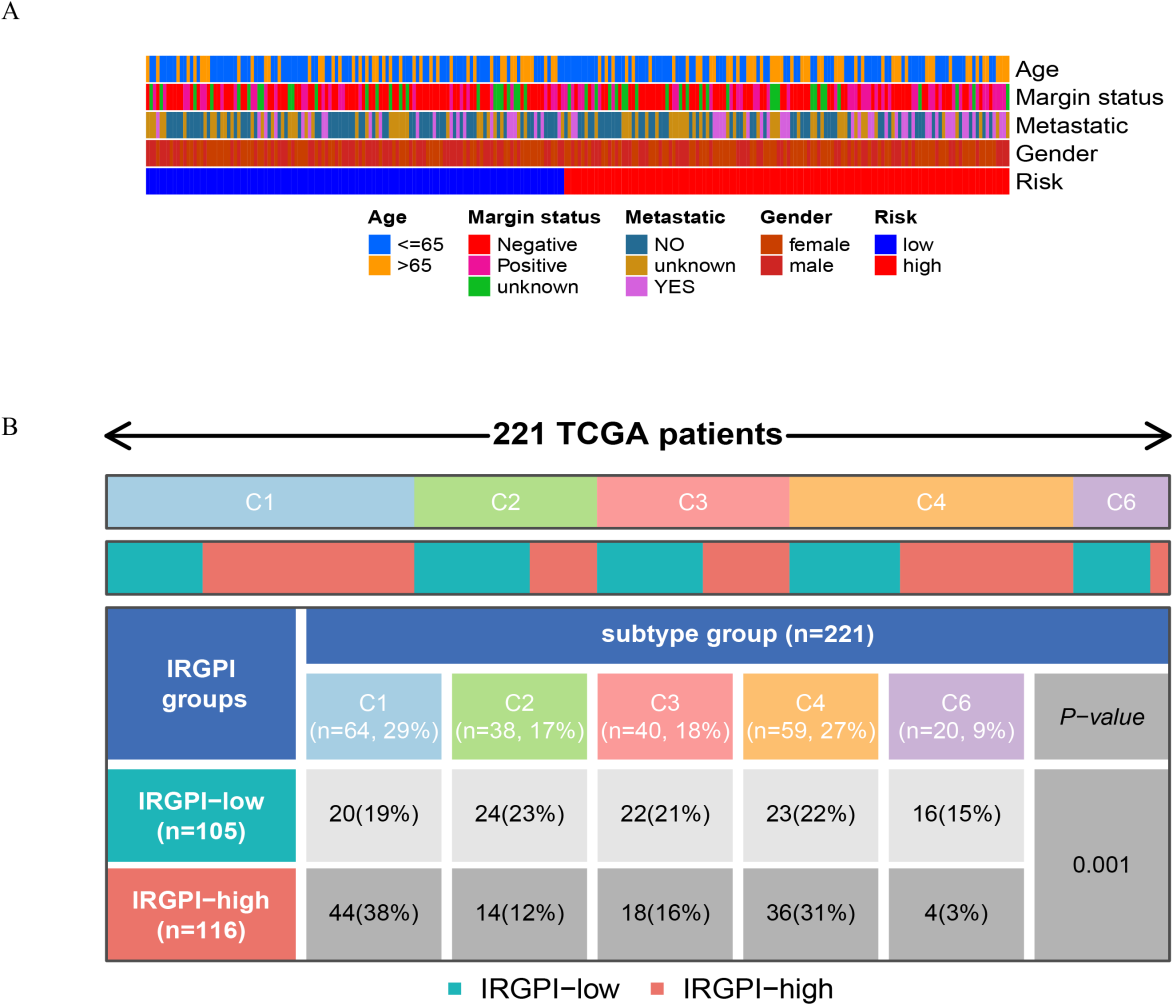
Relationship between IRGPM grouping and other immune and molecular subtypes. **(A)** The IRGPM grouping and proportions of 263 patients in TCGA cohort. Age, margin status, metastatic, and gender are shown as patient annotations. **(B)** The IRGPM subgroups are displayed in the heatmap, indicating the distribution of STS immune subtypes (C1,2,3,4,6).

To identify enriched gene sets in different subgroups of IRGPM, a GSEA was conducted. It was discovered that the gene collections in samples characterized by IRGPM-high were enriched in GO, such as chromosome segregation, DNA conformation and change, microtubule-cytoskeleton-organization, muscle and organ development, contractile fiber (**Figure 6A**); KEGG, such as cardiac muscle contraction, cell cycle, dilated cardiomyopathy, homologous recombination, TGF beta signaling pathway (**Figure 6C**), while the gene collections of the IRGPM-low group exhibited an enrichment in GO, such as adaptive immune response, activation of immune response, antigen receptor mediated signaling pathways (**Figure 6B**); KEGG, such as cytokine-cytokine receptor interaction, hematopoietic cell lineage, T cell receptor signaling pathway (**Figure 6D**). Next, for a deeper understanding of IRGPM subgroups’ somatic mutations, we analyzed gene mutations. It was discovered that the IRGPM high group had considerably more mutations compared to the IRGPM low group. Mutations with missense are the most common, followed by deletions with frameshifts and nonsense mutations. Afterwards, the IRGPM subgroups were analyzed to identify the top 20 genes with the highest mutation rates. It was discovered that the mutation rates of TP53, ATRX, and TTN surpassed 10% in both groups (**Figures 6E,F**). The IRGPM grouping and proportions such as age, margin status, metastatic, and gender of 256 patients in TCGA cohort were shown in (**Figure 7A**). Based on TCGA database information on the microenvironment and mutations, tumors were divided into six types: C1 (wound healing), C2 (interferon-γ-dominant), C3 (inflammatory), C4 (lymphocyte depleted), C5 (immunologically quiet), and C6 (TGF-βdominant) (9). Following that, the distribution of immune subtypes within the IRGPI subgroups was analyzed. In this study, the IRGPI-low subgroup consisted 19%C1, 23%C2, 21%C3, 22%C4 and 15%C6 samples, while the IRGPM-high subgroup consisted 38%C1, 12%C2, 16%C3, 31%C4 and 3%C6 samples. The number of C1 and C4 samples in the IRGPM high subgroup was significantly higher than that in the IRGPM low subgroup (P < 0.001). (**Figure 7B**).

### 3.4 Comprehensive analysis of tumor immune microenvironment in different IRGPM subgroups

**Figure 8:**
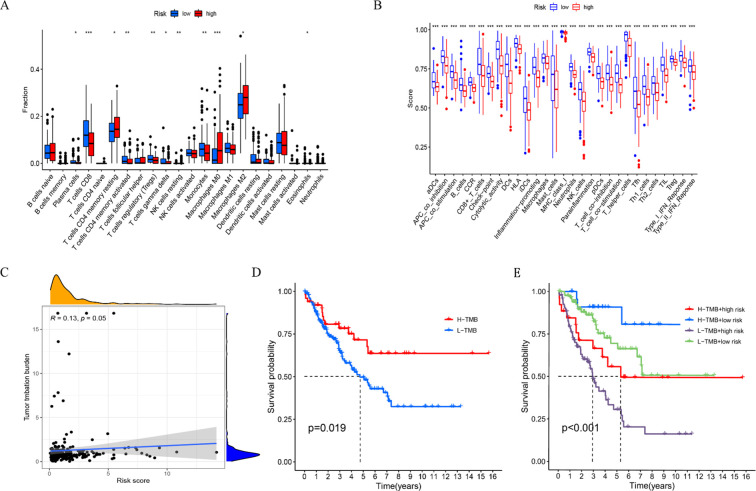
Comparison of immune features and TMB between high- and low-risk groups. **(A)** The proportion of immune cell types differed between the high and low risk groups among all patients. **(B)** The proportion of immune functions differs between the high risk group and low risk group. **(C)** Correlations between the TMB and risk scores in our proposed model. **(D)** The K-M curves of patients with high TMB and low TMB. **(E)** The K-M curves for patients with high TMB and low TMB in both the high risk and low risk groups. *P < 0.05, **P < 0.01, ***P < 0.001.

**Figure 9:**
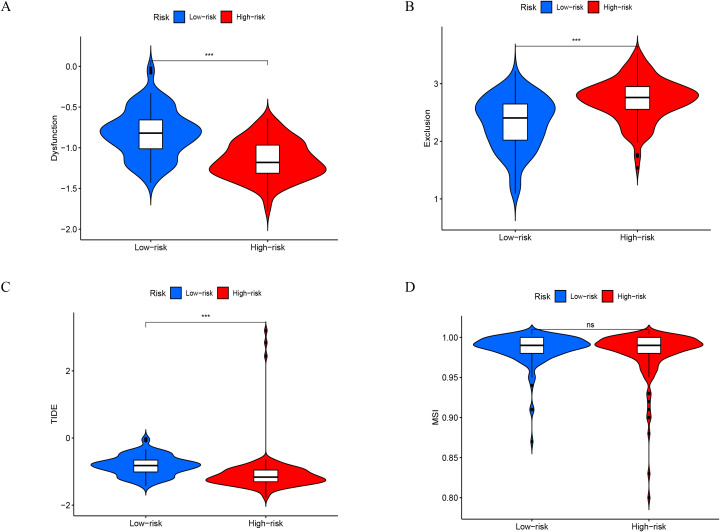
Immunotherapy response between high- and low-risk groups. **(A-D)** A violin plot reveals the contrast in dysfunction score, exclusion score, TIDE, and MSI among the high risk and low risk groups. ***P < 0.001.

We analyzed the composition of immune cells in various IRGPM subgroups by comparing their distribution using the Wilcoxon test. We found that M1 macrophages, in the IRGPM high subgroup, there were more T cells CD4 memory resting and Macrophages M2, whereas in the IRGPM low subgroup, there were more T cells with CD8 phenotype, Monocytes and T cells with regulatory phenotype (Tregs) (**Figure 8A**). Our next step was to apply certain gene model to determine immune functions between different subgroups of IRGPM. As a result, we found that most immune and molecular functions such as aDCs, APC_co_inhibition, APC_co_stimulation, B_cells, CCR, CD8+_T_cells, Check−point, Cytolytic_activity significantly increased in the IRGPM-low (**Figure 8B**). As a next step, we investigated the relationship between TMB and IRGPM score. There was a slight relation between IRGPM and TMB as a consequence (**Figure 8C**). We compared K-M curves for H TMB patients versus L TMB patients and for H TMB patients versus L TMB patients in high risk and low risk groups. According to the results, patients who had a higher TMB had a greater probability of survival, whereas patients with higher TMB who were at low risk had the greatest likelihood of survival. (**Figures 8D, E**). After that, we used TIDE to determine whether immunotherapy would be effective in different IRGPM subgroups. A higher potential for immune evasion was indicated by an increase in TIDE prediction score, which indicated a lower likelihood of immunotherapy benefiting the patients. Our results indicate that immunotherapy may be more advantageous for IRGPM high patients since the TIDE score of the IRGPM high subgroup was found to be lower than that of the IRGPM low subgroup (**Figures 9A–D**).

### 3.5 CALR expression is upregulated in different sarcoma cells compared to normal osteoblasts

**Figure 10:**
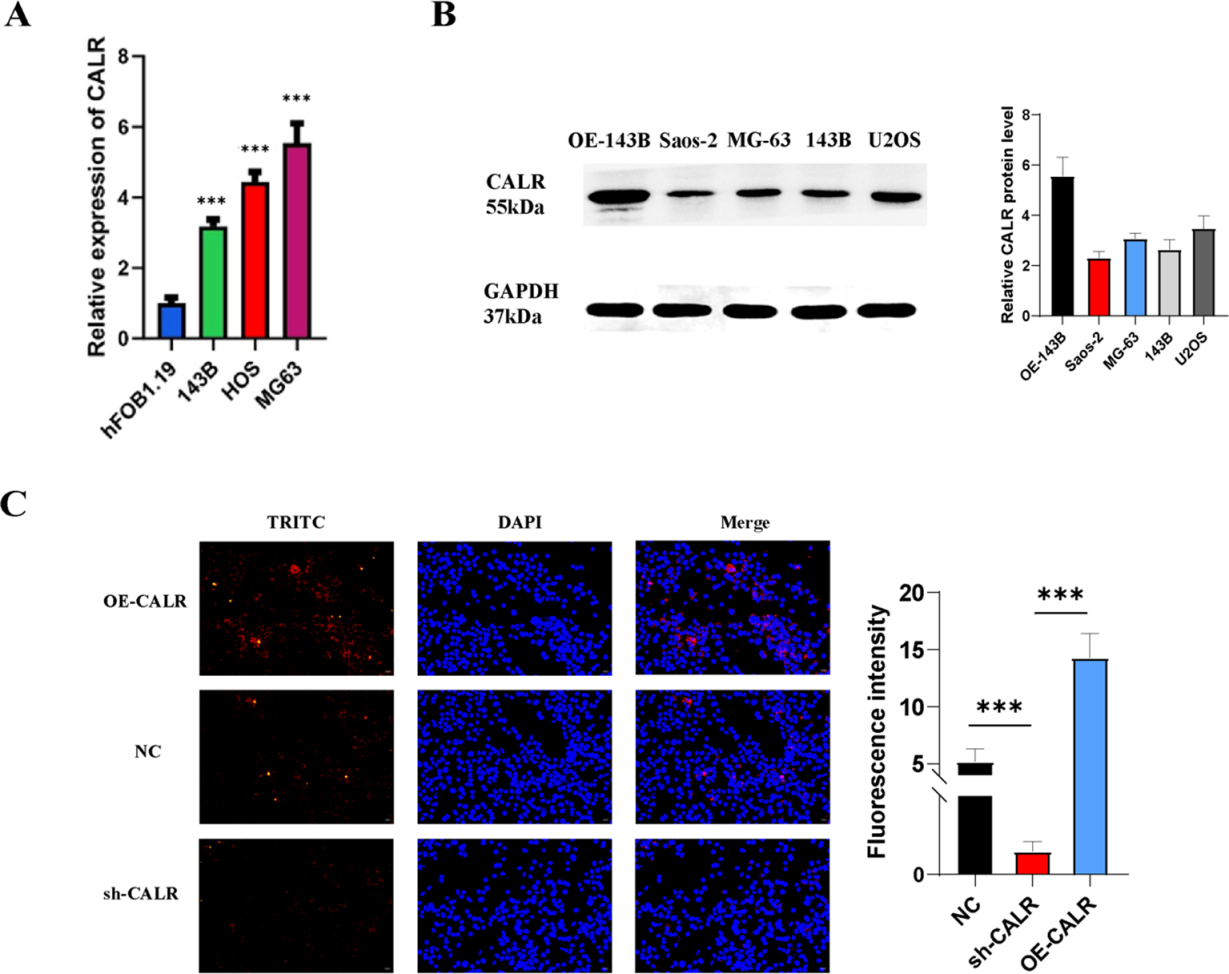
CALR is overexpressed in osteosarcoma and modulates cellular phenotypes. **(A)** qRT-PCR analysis of CALR mRNA levels in osteosarcoma cell lines (MG-63, 143B, HOS) compared to human normal osteoblasts (hFOB1.19). **(B)** Western blot showing endogenous CALR protein expression in untreated osteosarcoma cells (Saos-2, MG-63, 143B, U2OS) and CALR-overexpressing 143B cells (OE-CALR). **(C)** Immunofluorescence staining of CALR (TRITC, red) and nuclei (DAPI, blue) in 143B cells under overexpression (OE-CALR), knockdown (sh-CALR), and negative control (NC) conditions. Fluorescence intensity quantification is shown below. *P < 0.05, **P < 0.01, ***P < 0.001.

To determine the differential expression of CALR between sarcoma and normal cells, we performed qRT-PCR analysis on three sarcoma cell lines (MG-63, 143B, HOS) and the human normal osteoblast cell line hFOB1.19. As shown in **Figure 10A**, CALR mRNA levels were significantly elevated in all sarcoma cells compared to hFOB1.19 (p < 0.05 for MG-63, p < 0.01 for 143B and HOS; one-way ANOVA), indicating a consistent upregulation of CALR in malignant cells.

### 3.6 CALR protein levels are enhanced in overexpression models

Western blot analysis of untreated sarcoma cells (Saos-2, MG-63, 143B, U2OS) and CALR-overexpressing 143B cells (OE-CALR) revealed distinct expression profiles. While endogenous CALR protein (~55 kDa) was detectable at low levels in all untreated sarcoma cell lines, its expression was markedly increased in the OE-CALR group (**Figure 10B**). Densitometric quantification showed a 4.5-fold increase in CALR protein in OE-CALR compared to untreated 143B cells (p < 0.001, Student’s t-test). GAPDH (37 kDa) served as the loading control.

### 3.7 Subcellular localization and quantitative fluorescence analysis of CALR

Immunofluorescence staining in 143B cells further validated CALR modulation. Overexpression of CALR (OE-CALR) resulted in strong TRITC signals (red) localized predominantly in the endoplasmic reticulum, whereas CALR knockdown (sh-CALR) significantly reduced fluorescence intensity (**Figure 10C**). Quantitative analysis demonstrated a 3.1-fold increase in CALR fluorescence in the OE group and a 68% reduction in the sh-CALR group compared to the NC control (p < 0.001, **Figure 10C**, lower panel). Nuclear staining with DAPI (blue) confirmed preserved cellular architecture across all groups.

## 4 Discussion

With the emergence of targeted therapy and immunotherapy in the field of tumor treatment, chemotherapy is still the cornerstone of STS treatment (10). The timing, indications and protocol selection of chemotherapy need to be combined with the treatment purpose and the chemotherapy sensitivity and risk of the tumor as well as the general condition of the patient. To enhance the chemotherapeutic efficacy of STS, the combined application of chemotherapy and targeted therapy or immunotherapy will become an important direction for the next research (11, 12). As a result, we conducted a study that emphasized the diversity of STS and the relationship between infiltration of immune cells in tumors and tumor cells, as this has immense importance in comprehending the mechanisms of tumor growth and predicting prognosis. Additionally, these findings may lead to the identification of novel diagnostic and treatment methods. We selected qRT-PCR, Western blotting, and immunofluorescence staining as validation techniques based on their complementary strengths. qRT-PCR was used to assess mRNA expression levels of CALR in tumor versus normal cells, while Western blotting confirmed protein expression. Immunofluorescence further visualized subcellular localization and relative abundance of CALR in transfected cells. These three approaches together provided robust and multi-dimensional validation of the CALR expression profile predicted by our bioinformatics analyses. Our study identified and validated the 8 immune related gene prognostic model (IRGPM) using mRNA sequencing data from TCGA and GTEx. Using the risk score derived from our IRGPM, we categorized the patients into high-risk and low-risk subgroups. Our next step was to comprehensively analyze the tumor immune microenvironment features of our subgroups. As we know, tumorigenesis and progression are not only related to its own characteristics, but also influenced by the tumor microenvironment (TME) in which it is located (13, 14). TME is consisted of stromal cells (fibroblasts, macrophages, endothelial cells, etc.), ECM components (inflammatory cytokines, chemokines, etc.) and exosomes (extracellular vesicles containing small molecules), which play an important role in tumor development (15, 16). According to the literature, tumor-associated macrophages in stromal cells, i.e., M2 macrophages, can promote angiogenesis and stromal remodeling and are closely linked to the progression and prognosis of STS (17–19). Our study revealed that the IRGPI high subgroup exhibited a greater abundance of M1 macrophages, T cells CD4 memory resting, and Macrophages M2, whereas the IRGPI low subgroup showed a higher presence of T cells CD8, Monocytes, and T cells regulatory (Tregs). Later, different IRGPM subgroups were identified for enriched gene sets through GSEA analysis. The enrichment of the gene sets in GO was discovered in samples with IRGPM high, such as chromosome segregation, DNA conformation and change, microtubule-cytoskeleton-organization, muscle and organ development, contractile fiber; KEGG, such as cardiac muscle contraction, cell cycle, dilated cardiomyopathy, homologous recombination, TGF beta signaling pathway, while the gene sets in the IRGPM samples with low expression showed enrichment in GO, such as activation of immune response, adaptive immune response, antigen receptor mediated signaling pathways; KEGG, such as cytokine-cytokine receptor interaction, hematopoietic cell lineage, T cell receptor signaling pathway. Next, for a deeper understanding of IRGPM subgroups’ somatic mutations, we analyzed gene mutations. It was discovered that there was a significant increase in mutations in the IRGPM high group compared to the IRGPM low group. Mutations with missense are the most common, followed by deletions with frameshifts and nonsense mutations. Finally, in the IRGPM subgroups, the 20 genes showing the greatest mutation frequencies were singled out. In both groups, TP53, ATRX, and TTN had mutation rates exceeding 10%. Six distinct molecular subtypes have been consistently reported (9, 20). Following that, the distribution of immune subtypes within the IRGPM subgroups was analyzed. In this study, the IRGPM-low subgroup consisted 19%C1, 23%C2, 21%C3, 22%C4 and 15%C6 samples, while the IRGPM-high subgroup consisted 38%C1, 12%C2, 16%C3, 31%C4 and 3%C6 samples. There were a greater number of C1 and C4 samples found in the IRGPM high subgroup compared to the IRGPM low subgroup. Next, we explore the correlation of TMB and IRGPM score. There was a slight relation between IRGPM and TMB as a consequence. We compared K-M curves for H TMB patients versus L TMB patients and for H TMB patients versus L TMB patients in high risk and low risk groups. According to the results, patients who had a higher TMB had a greater probability of survival, whereas patients with higher TMB who were at low risk had the greatest likelihood of survival, which were consistent with situation in various cancers (21–25). After that, we used TIDE to determine whether immunotherapy would be effective in different IRGPM subgroups. A higher potential for immune evasion was indicated by an increase in TIDE prediction score, which indicated a lower likelihood of immunotherapy benefiting the patients (26–28). Based on our results, the IRGPM low subgroup had a higher TIDE score than the IRGPM high subgroup, suggesting that immunotherapy would be more beneficial to IRGPM-high patients. In recent years, new technologies, methods, and drugs for immunotherapy are rapidly emerging, bringing new opportunities for the treatment of STS, which has been stagnant for many years. However, while several basic studies have suggested that immunotherapy can provide additional clinical benefits for patients with STS, the results of clinical trials conducted with single therapies are often unsatisfactory, making it difficult to achieve a comprehensive and effective treatment for STS (29). Several high-profile star drugs have faltered in the treatment of STS (30, 31). The reasons for this are that most of the cases included in these clinical trials are relapsed, progressive and metastatic advanced STS after conventional chemotherapy, and their immune systems are often severely destroyed and difficult to achieve good remodeling, which significantly confine the immunotherapy’s efficacy. In addition, the immune microenvironment of tumor patients is often complex and variable, and tumor cells are adept at using multiple pathways to resist immunotherapy, making it difficult to achieve success with a single immunotherapy. A number of clinical trials based on this have achieved good results (32–34). At the same time, the challenges of personalized selection of immunotherapy, immunotherapy resistance, and drug toxicity in combination therapy should be further addressed.

### 4.1 Limitations

Several limitations should be admitted in our study. First, there are insufficient samples and some other clinical pathological characteristics. Secondly, our prognostic model needs additional STS datasets to validate its performance. Last but not least, although CALR have been confirmed from practical experiments, they require confirmation through cell variability, and proliferation experiments.

## 5 Conclusions

IRGPM is a promising immune-related prognostic biomarker. As a prognostic indicator of immunotherapy, IRGPM might also help differentiate molecular and immune characteristics in STS.

## Data Availability

Publicly available datasets were analyzed in this study. This data can be found here: The Cancer Genome Atlas (TCGA)-STS and The Genotype-Tissue Expression (GTEx) database (https://xenabrowser.net/datapages/) were used to download RNA sequencing data from 392 samples, including 263 STS samples, 129 normal samples as well as their clinicopathologic characteristics. The ImmPort (https://www.immport.org/shared/home) and InnateDB (https://www.innatedb.com/) databases were used to download immune-related gene lists.
