# Supplementary material for: Identification and comprehensive analysis of an immune-related gene prognostic model for indicating tumor immune microenvironment features in soft tissue sarcoma
Source: Front Oncol. 2025 Sep 3;15:1609501. doi: 10.3389/fonc.2025.1609501 (PMC12444014; doi:10.3389/fonc.2025.1609501)
Supplement: Supplementary file 1 [file DataSheet1.zip › Supplementary/Supplementary tables.docx]

**Supplementary table 1. A List of Primers Used in This Study.**

| Gene | Forward sequence (5’ to 3’) | Reverse sequence (5’ to 3’) |
| --- | --- | --- |
| GAPDH | GGAGCGAGATCCCTCCAAAAT | GGCTGTTGTCATACTTCTCATGG |
| CALR | CCTGCCGTCTACTTCAAGGAG | GAACTTGCCGGAACTGAGAAC |

**Supplementary table 2. A list of CALR shRNA and Scrambled shRNA.**

| Target | Company | Catalog # | shRNA Sequence (5'-3') | Vector |
| --- | --- | --- | --- | --- |
| CALR | Santa Cruz | sc-141687-SH | CTGAGGAGGAGGTGCTGAC | pLKO.1-puro |
| Scrambled | Santa Cruz | sc-108060 | CGTCTACTTCAAGGATTGCT | pLKO.1-puro |

**Supplementary table 3. A list of CALR Overexpression Plasmid.**

| Plasmid | Company | Catalog # | Insert Description | Vector |
| --- | --- | --- | --- | --- |
| CALR-OE | GeneCopoeia | EX-H0146-M61 | Human CALR (NM_004343.4) + Flag | pcDNA3.1 |
