# Supplementary figures and images for: Identification and comprehensive analysis of an immune-related gene prognostic model for indicating tumor immune microenvironment features in soft tissue sarcoma
Source: Front Oncol. 2025 Sep 3;15:1609501. doi: 10.3389/fonc.2025.1609501 (PMC12444014; doi:10.3389/fonc.2025.1609501)

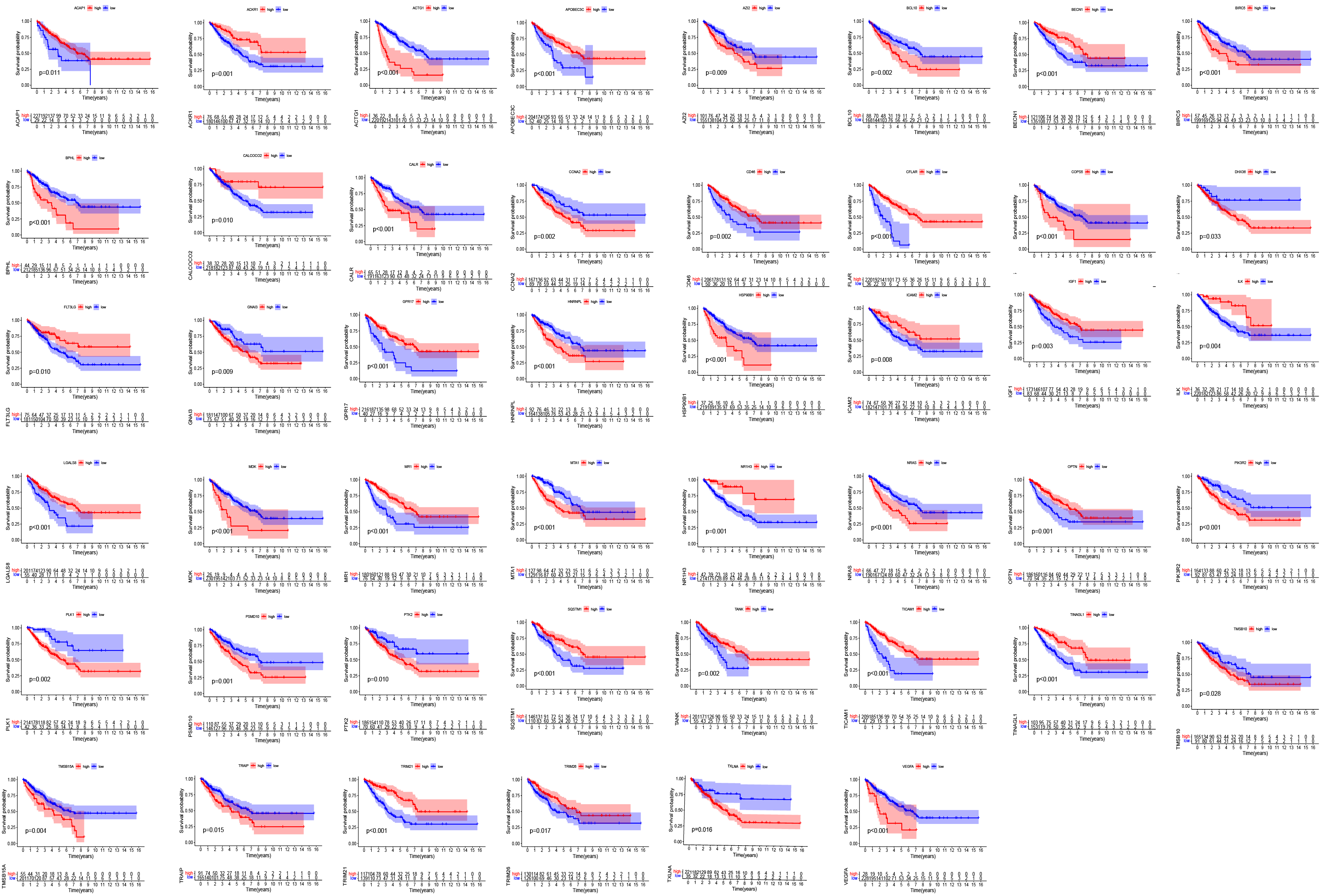

Supplement: Supplementary file 1 [file DataSheet1.zip › Supplementary/Supplementary Figure 1.tif]

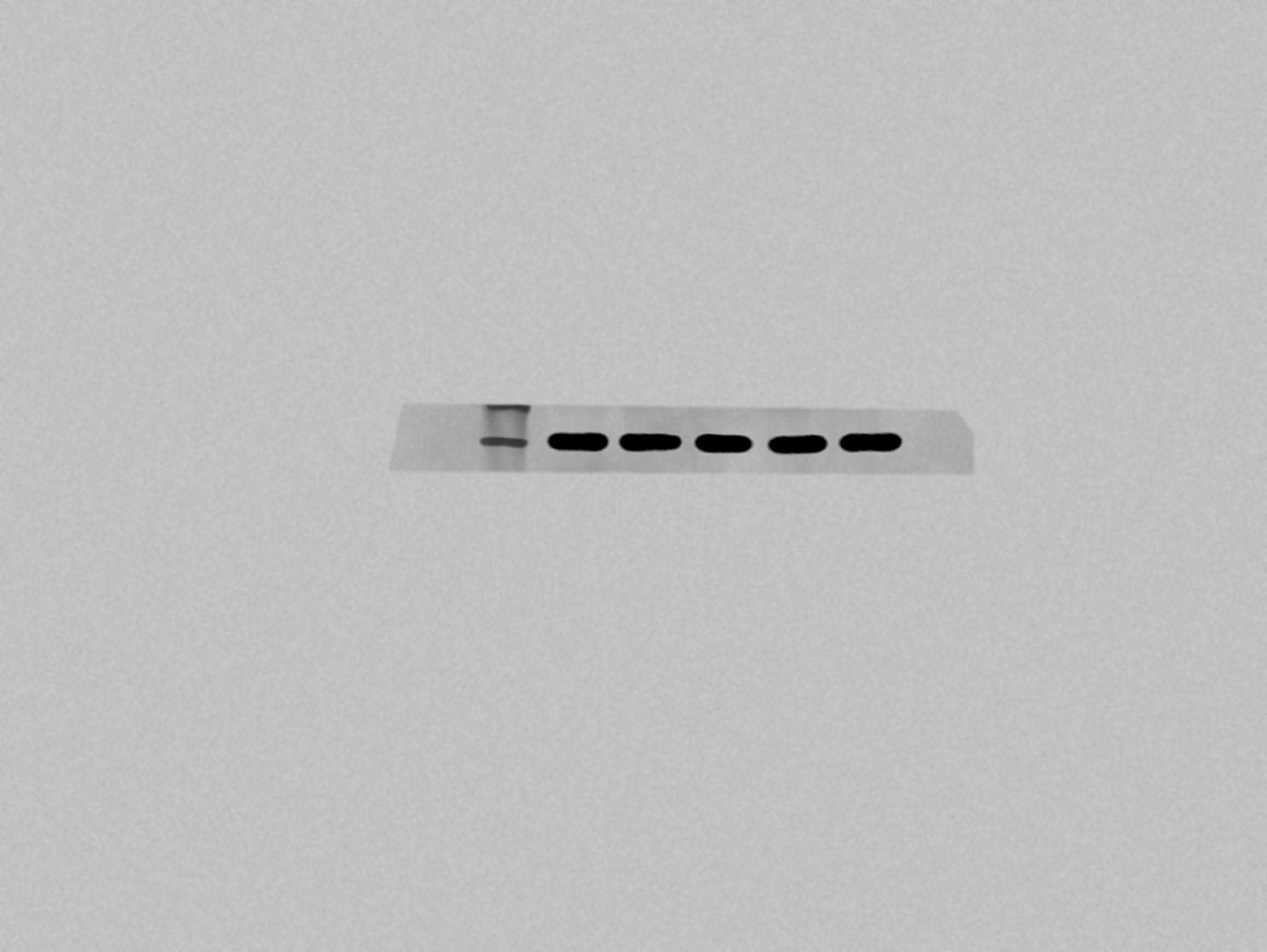

Supplement: Supplementary file 1 [file DataSheet1.zip › Supplementary/1-Western blot/GAPDH/GAPDH 1.tif]

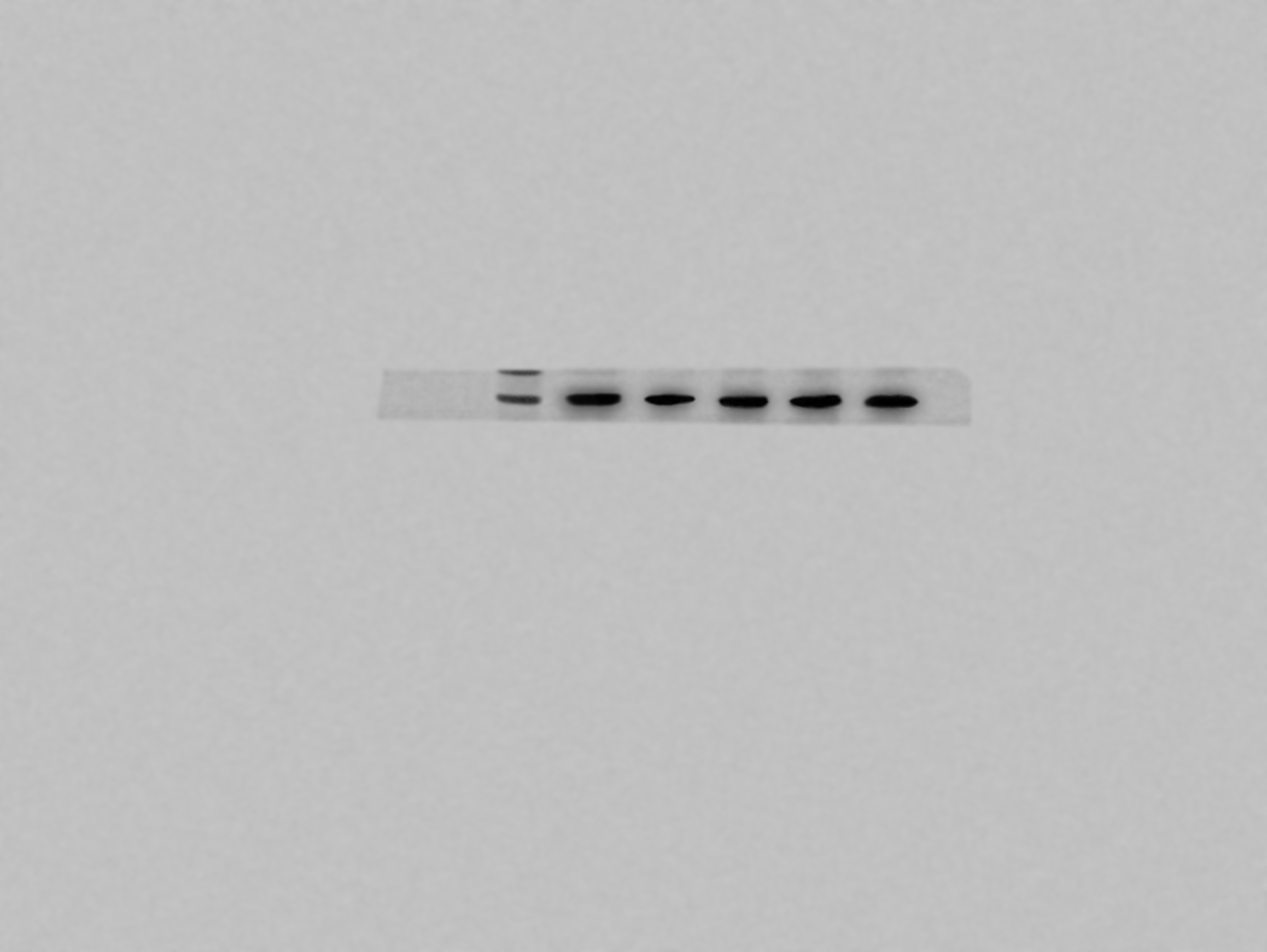

Supplement: Supplementary file 1 [file DataSheet1.zip › Supplementary/1-Western blot/GAPDH/GAPDH 2.jpg]

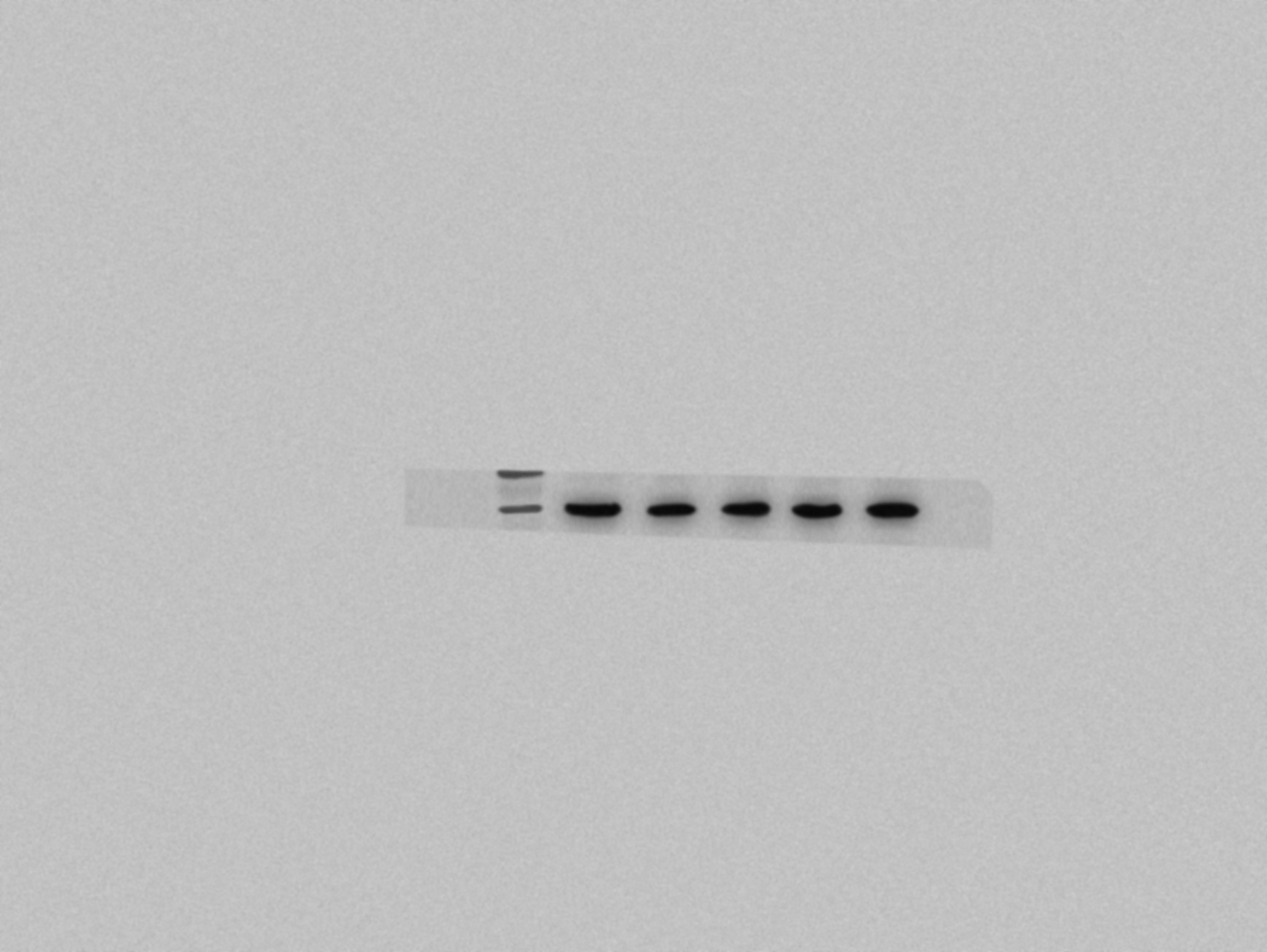

Supplement: Supplementary file 1 [file DataSheet1.zip › Supplementary/1-Western blot/GAPDH/GAPDH 3.jpg]

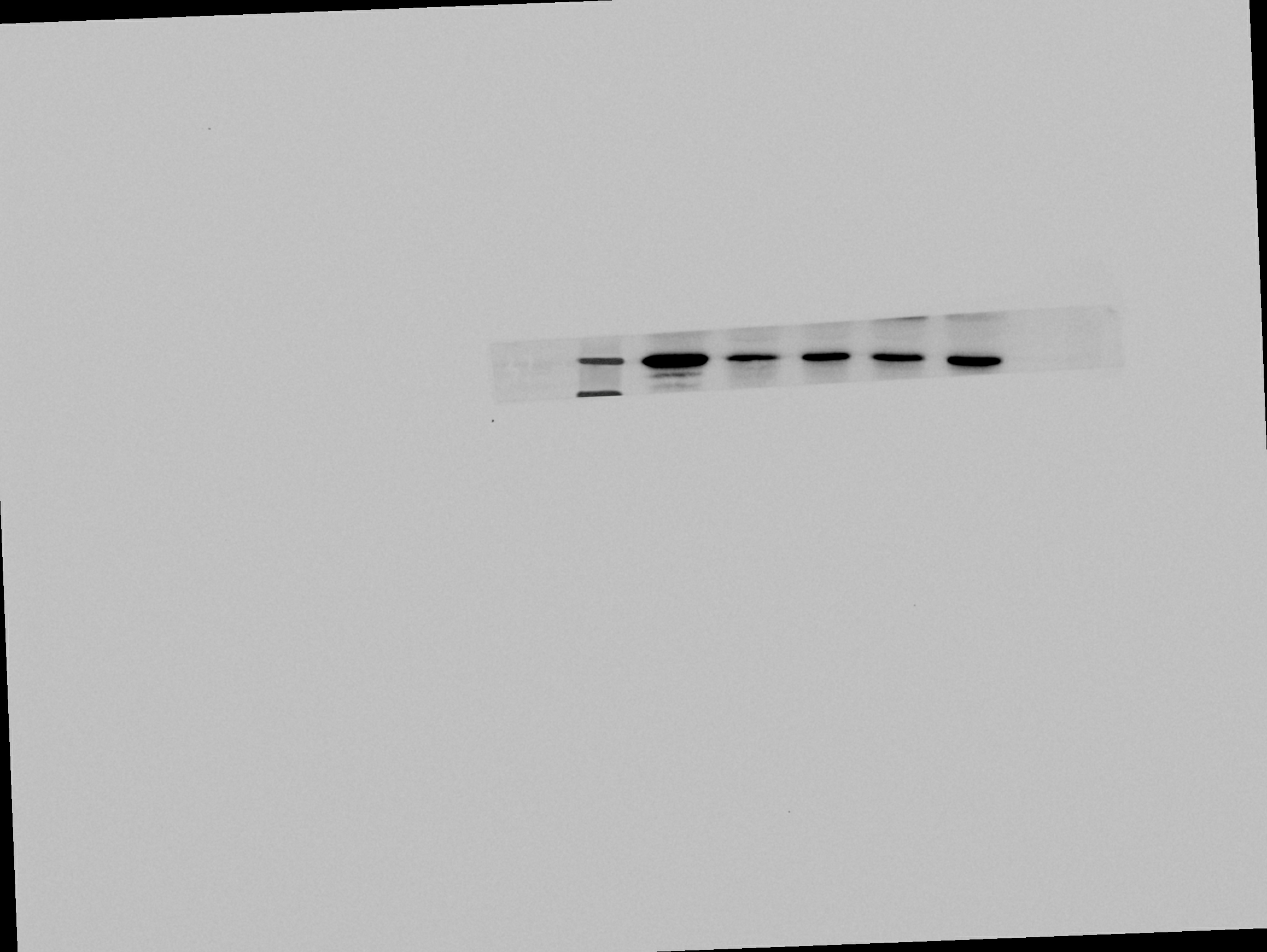

Supplement: Supplementary file 1 [file DataSheet1.zip › Supplementary/1-Western blot/Target/Target 1.tif]

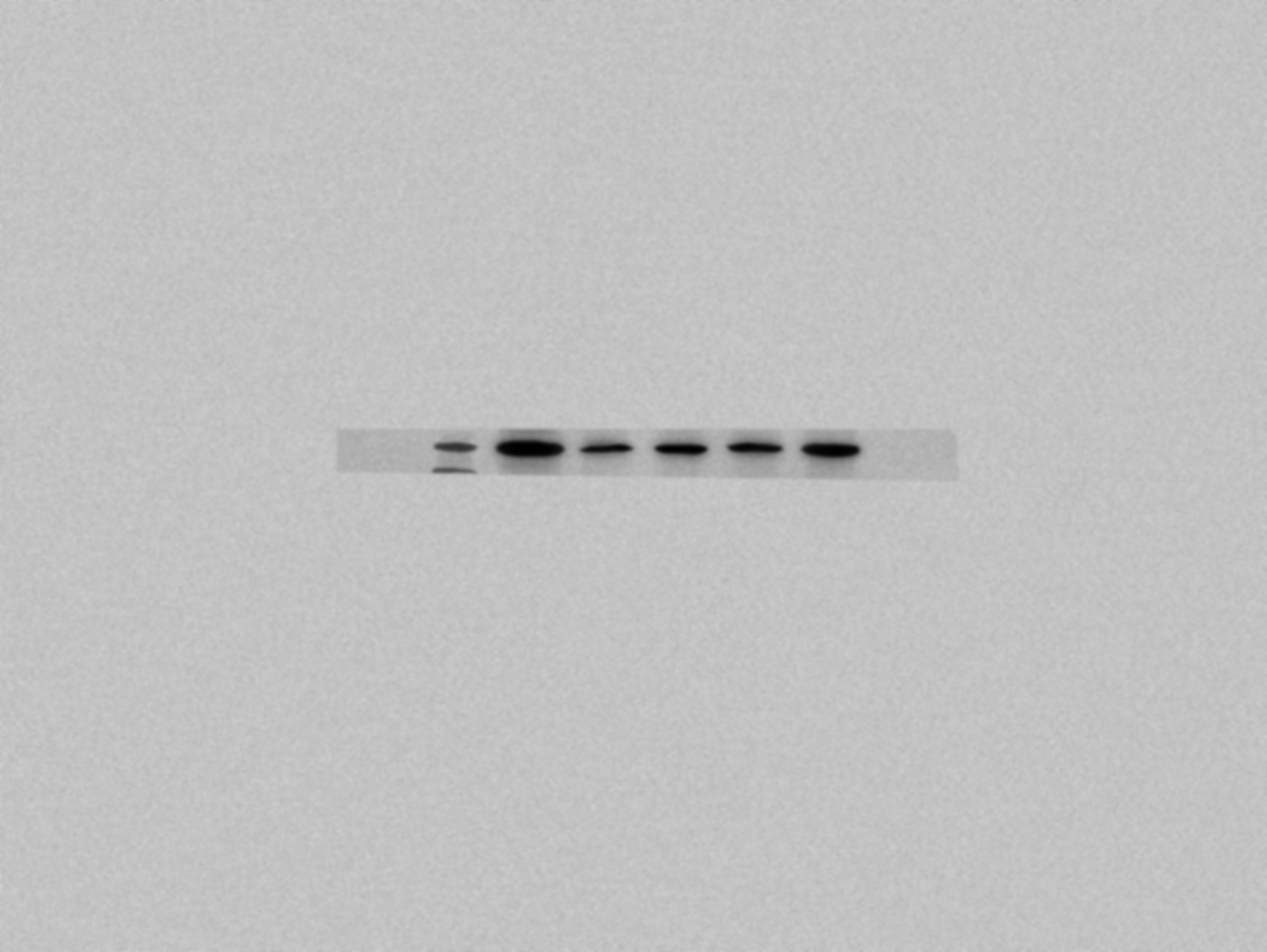

Supplement: Supplementary file 1 [file DataSheet1.zip › Supplementary/1-Western blot/Target/Target 2.jpg]

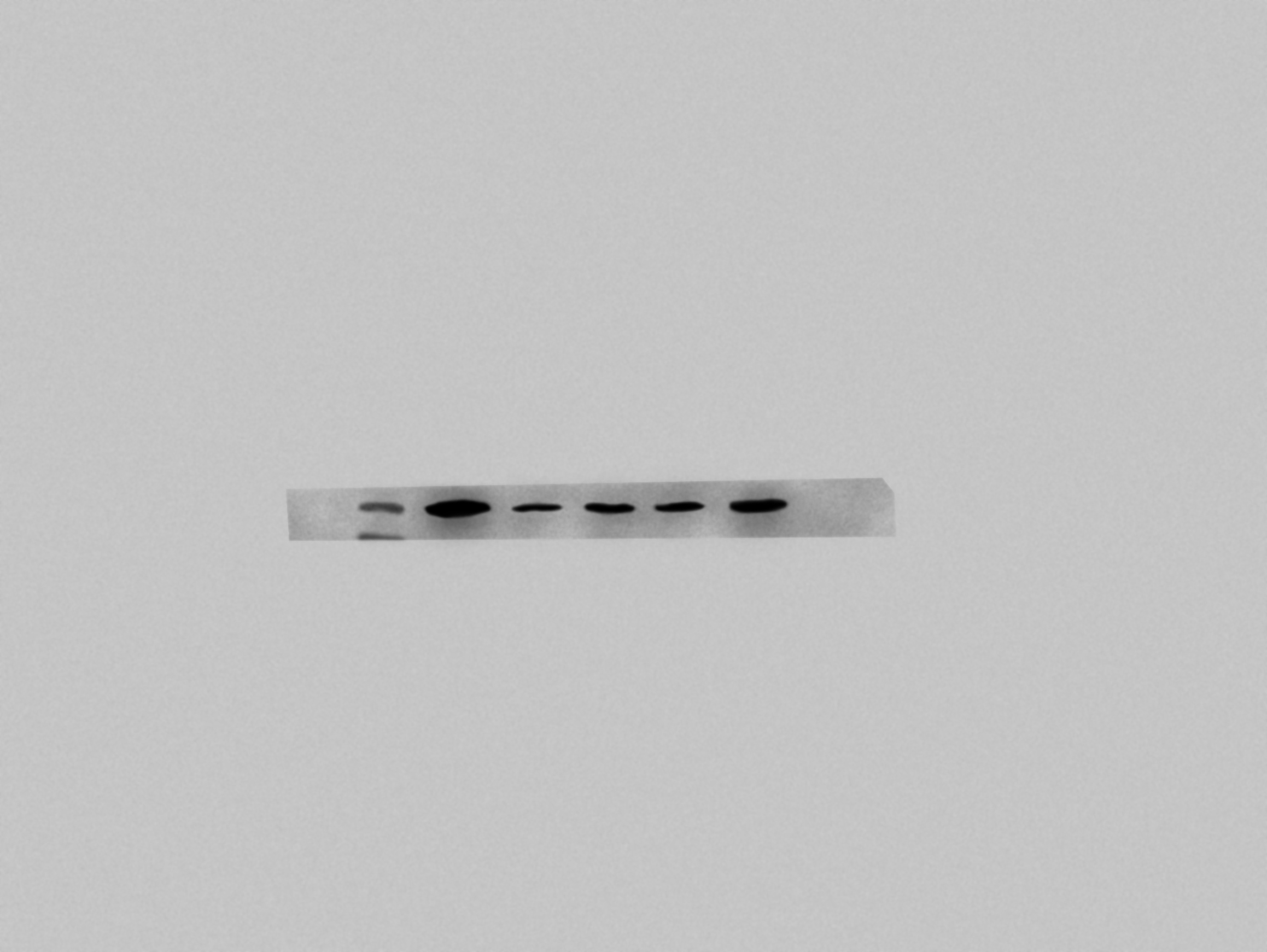

Supplement: Supplementary file 1 [file DataSheet1.zip › Supplementary/1-Western blot/Target/Target 3.jpg]

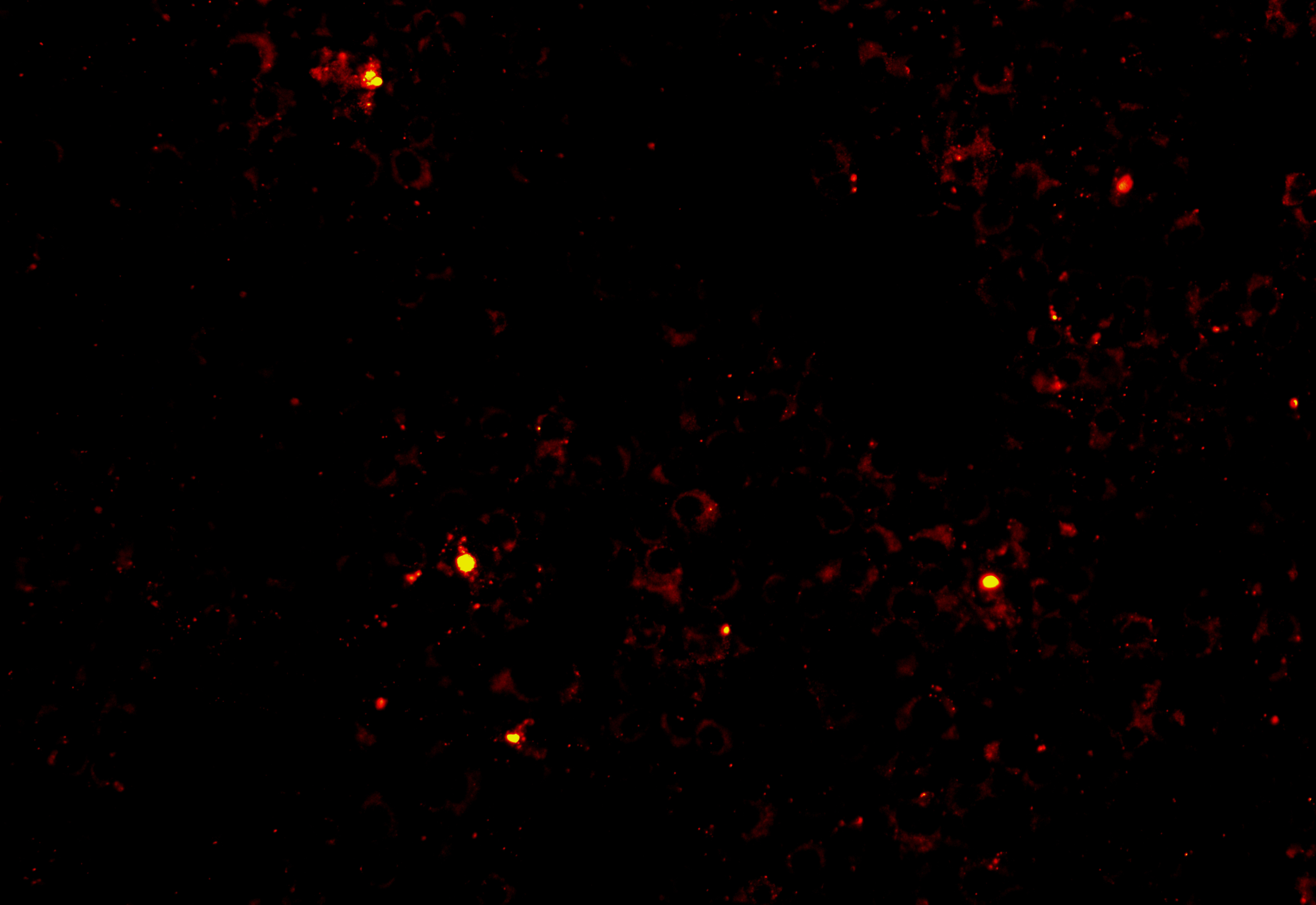

Supplement: Supplementary file 1 [file DataSheet1.zip › Supplementary/2-Immunofluorescence staining/NC group/1/1- 1.jpg]

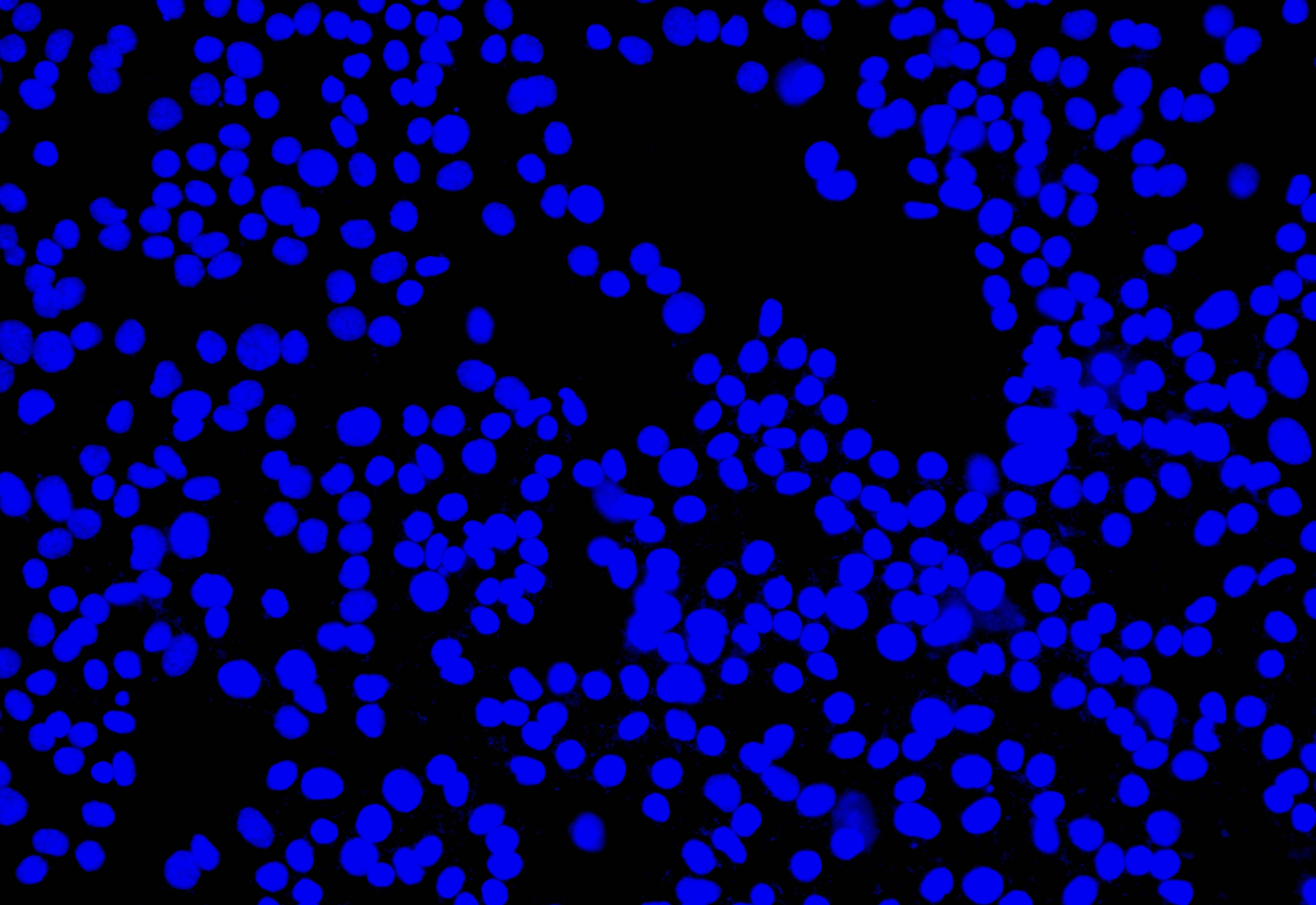

Supplement: Supplementary file 1 [file DataSheet1.zip › Supplementary/2-Immunofluorescence staining/NC group/1/1- 2.jpg]

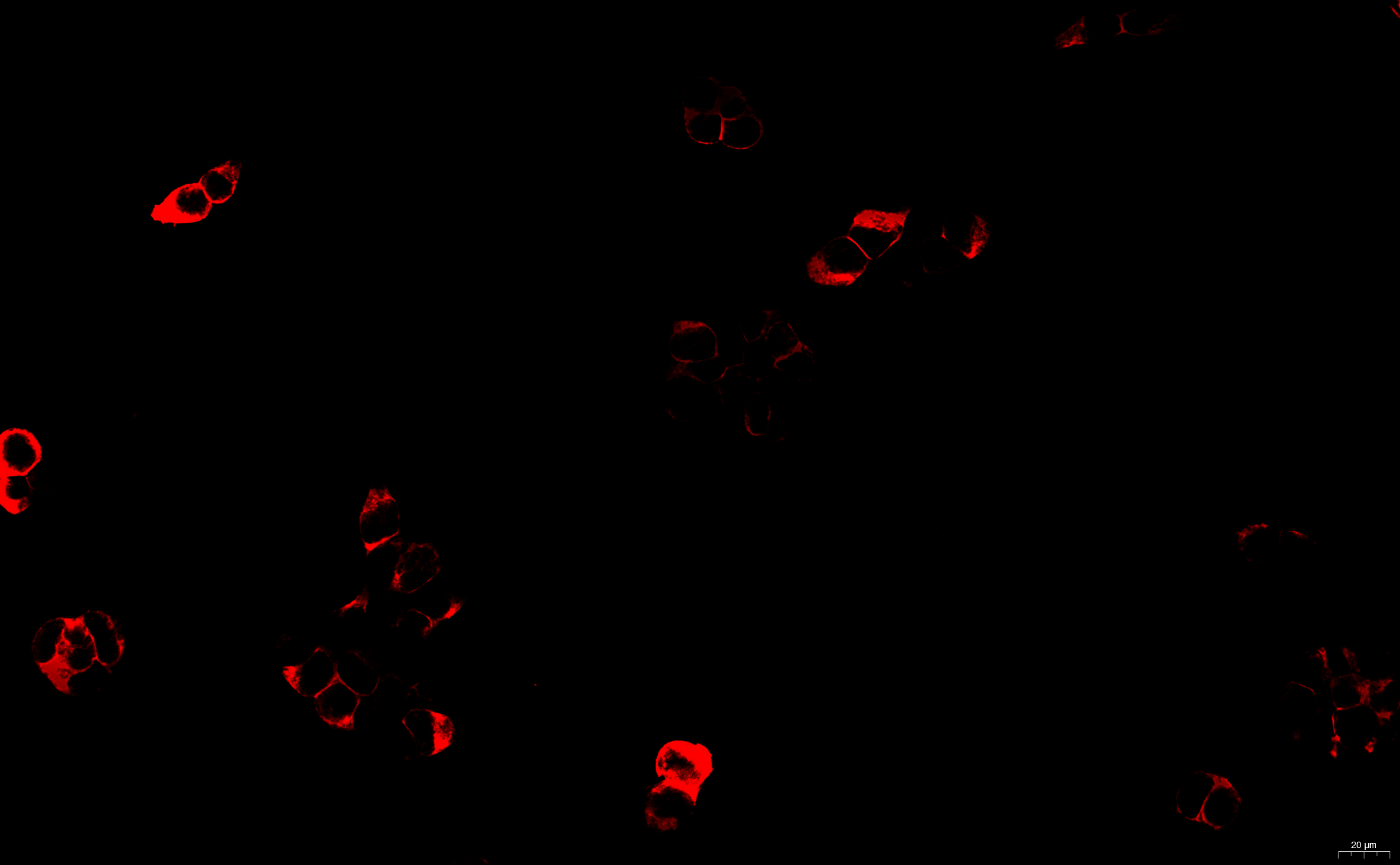

Supplement: Supplementary file 1 [file DataSheet1.zip › Supplementary/2-Immunofluorescence staining/NC group/2/1.tif]

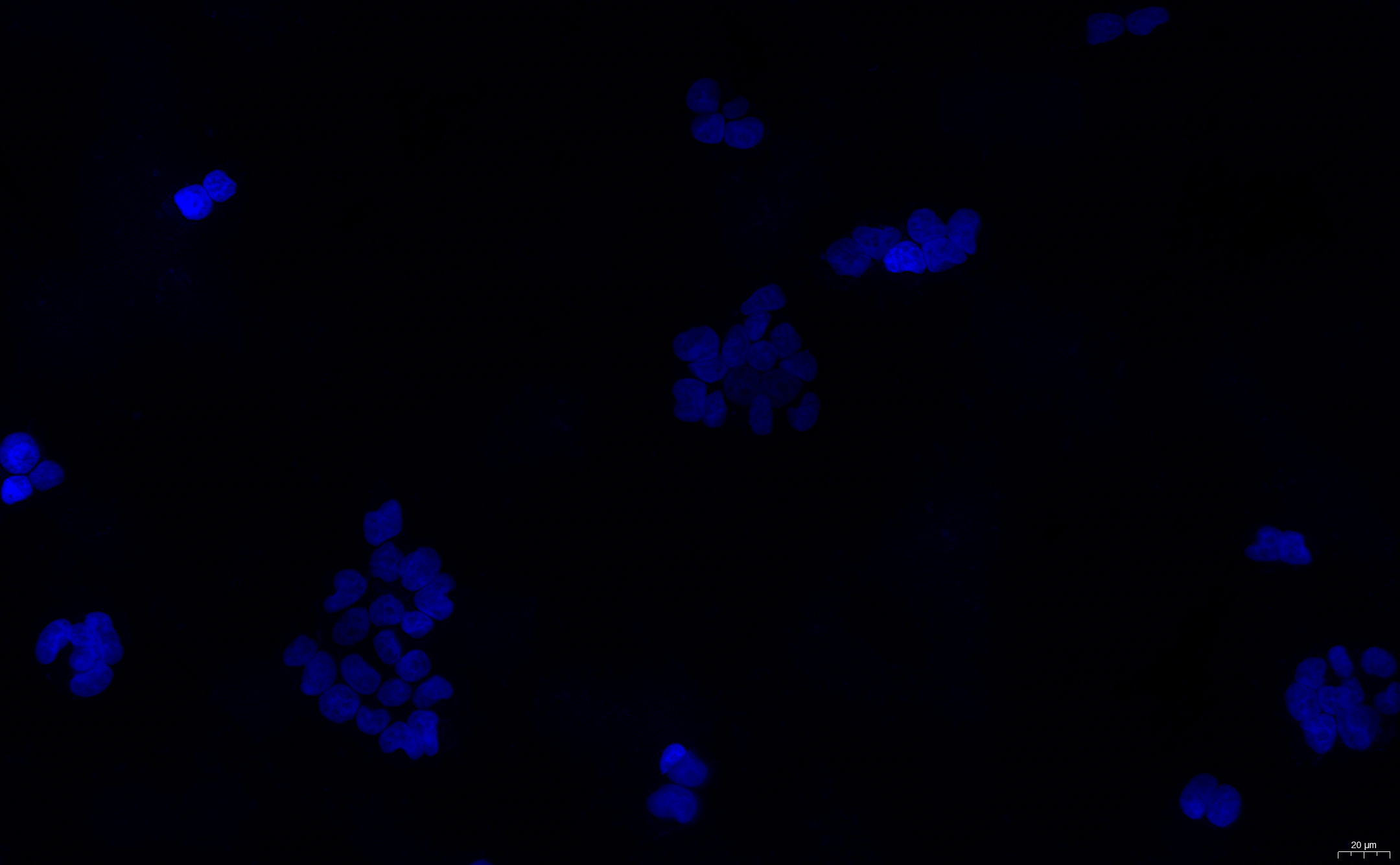

Supplement: Supplementary file 1 [file DataSheet1.zip › Supplementary/2-Immunofluorescence staining/NC group/2/2.tif]

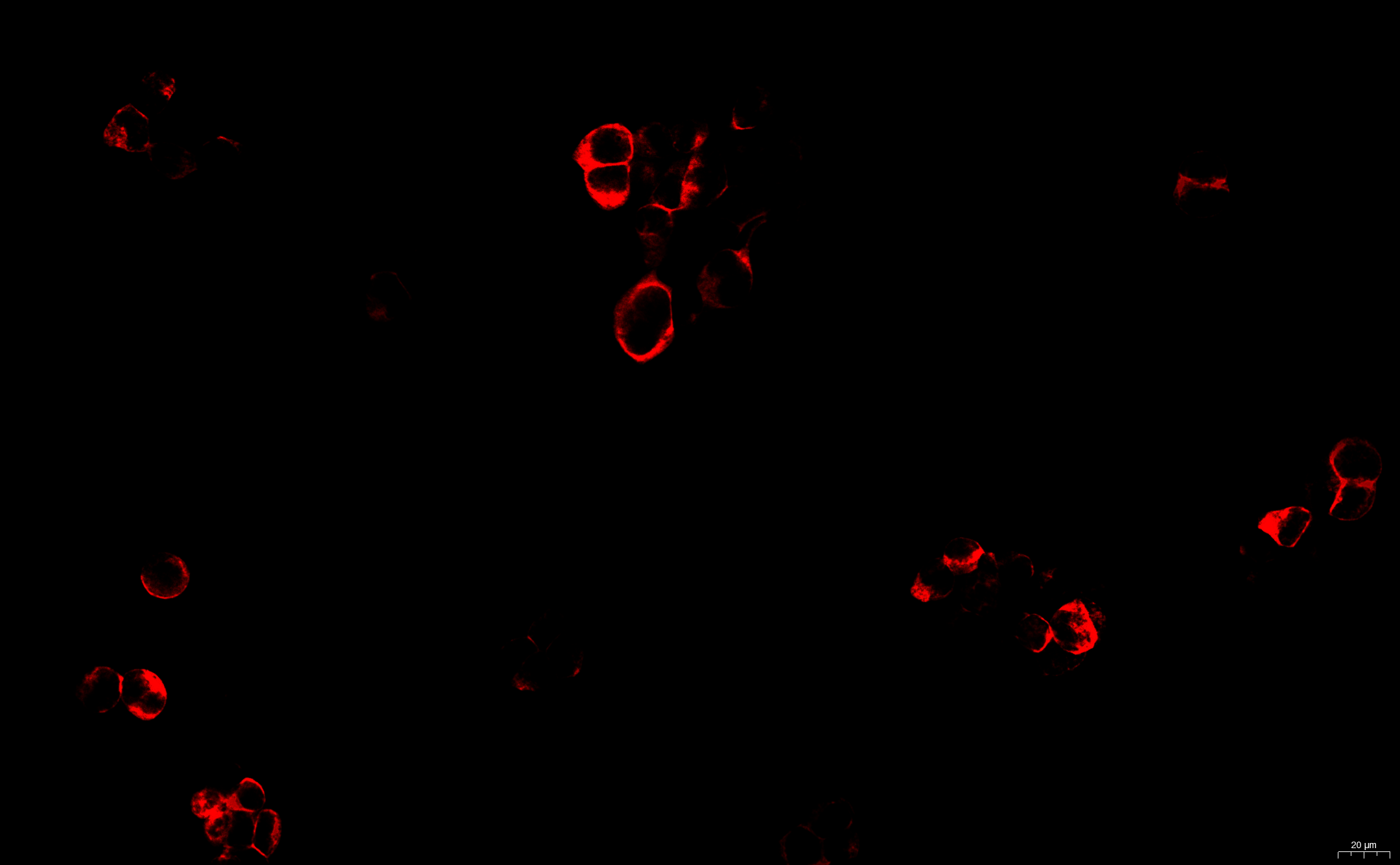

Supplement: Supplementary file 1 [file DataSheet1.zip › Supplementary/2-Immunofluorescence staining/NC group/3/1.tif]

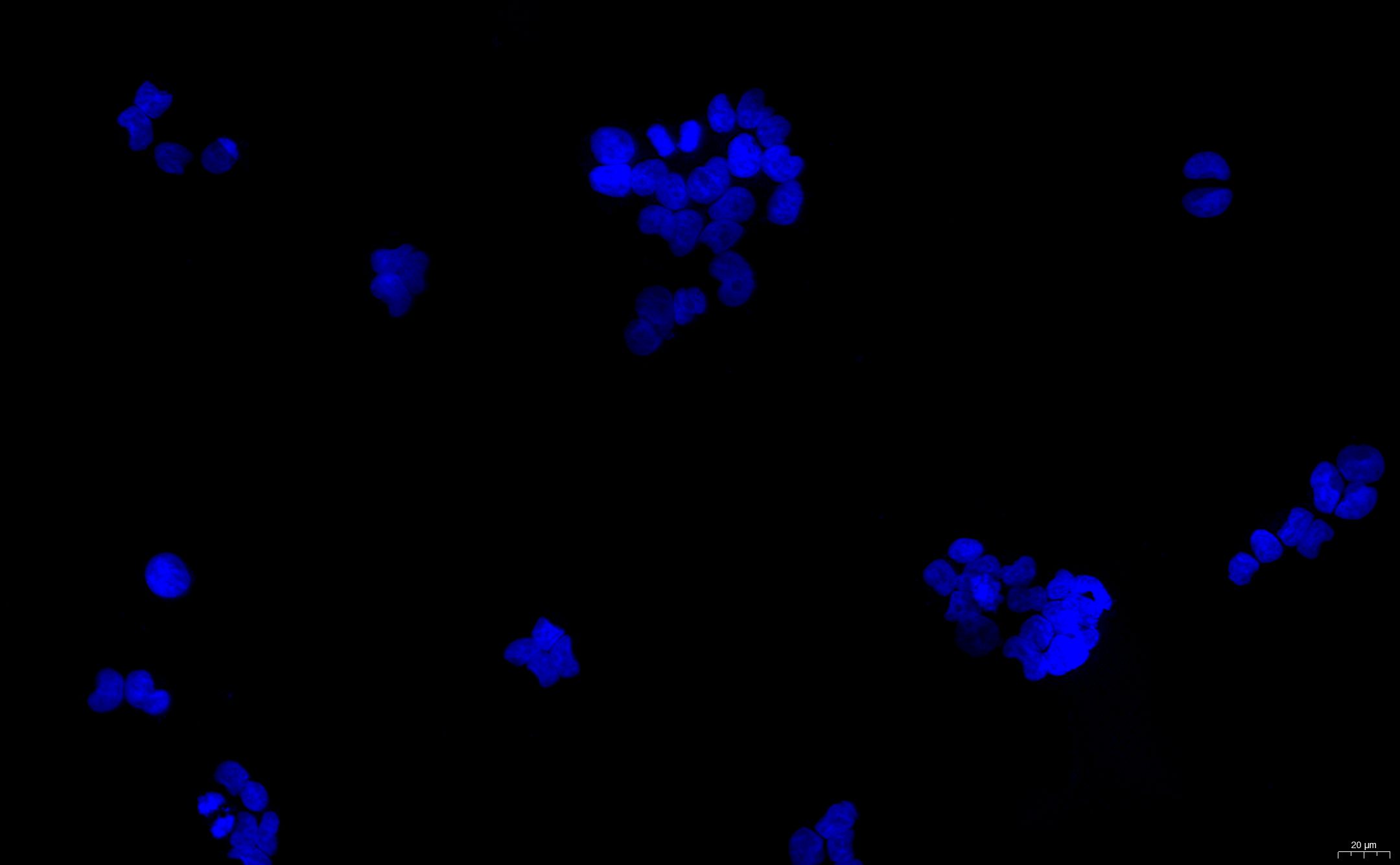

Supplement: Supplementary file 1 [file DataSheet1.zip › Supplementary/2-Immunofluorescence staining/NC group/3/2.tif]

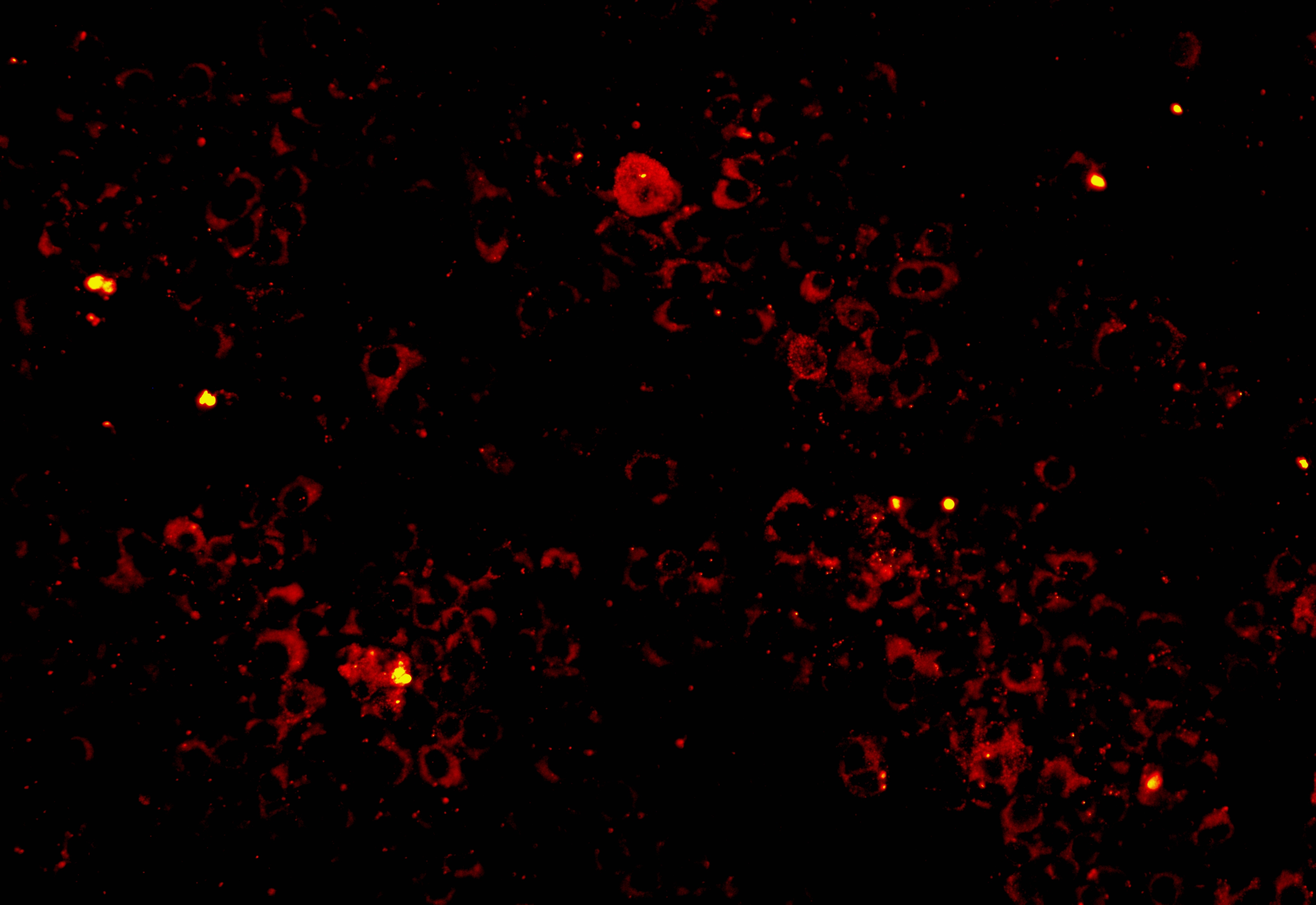

Supplement: Supplementary file 1 [file DataSheet1.zip › Supplementary/2-Immunofluorescence staining/OE group/1/1-1.jpg]

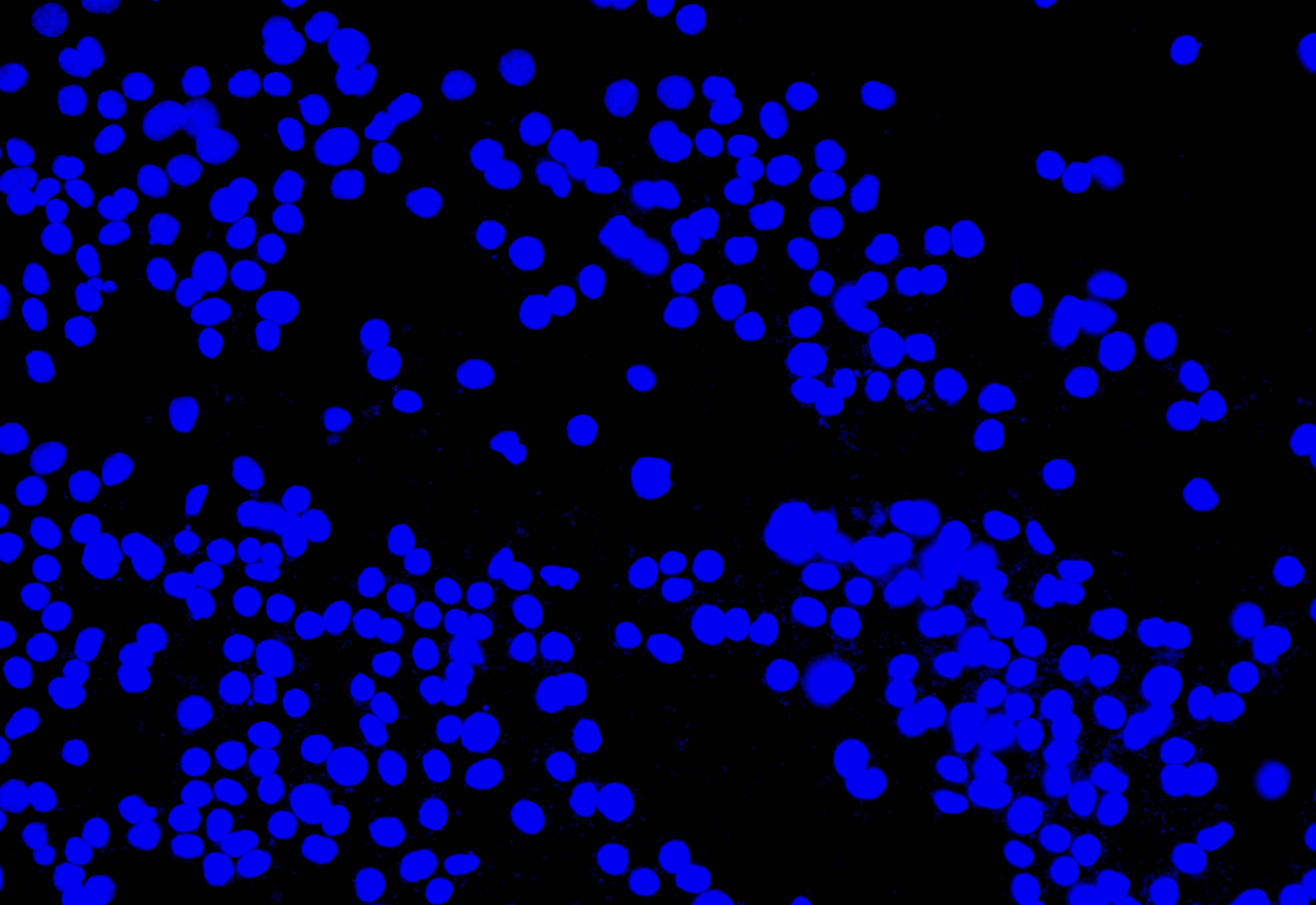

Supplement: Supplementary file 1 [file DataSheet1.zip › Supplementary/2-Immunofluorescence staining/OE group/1/1-2.jpg]

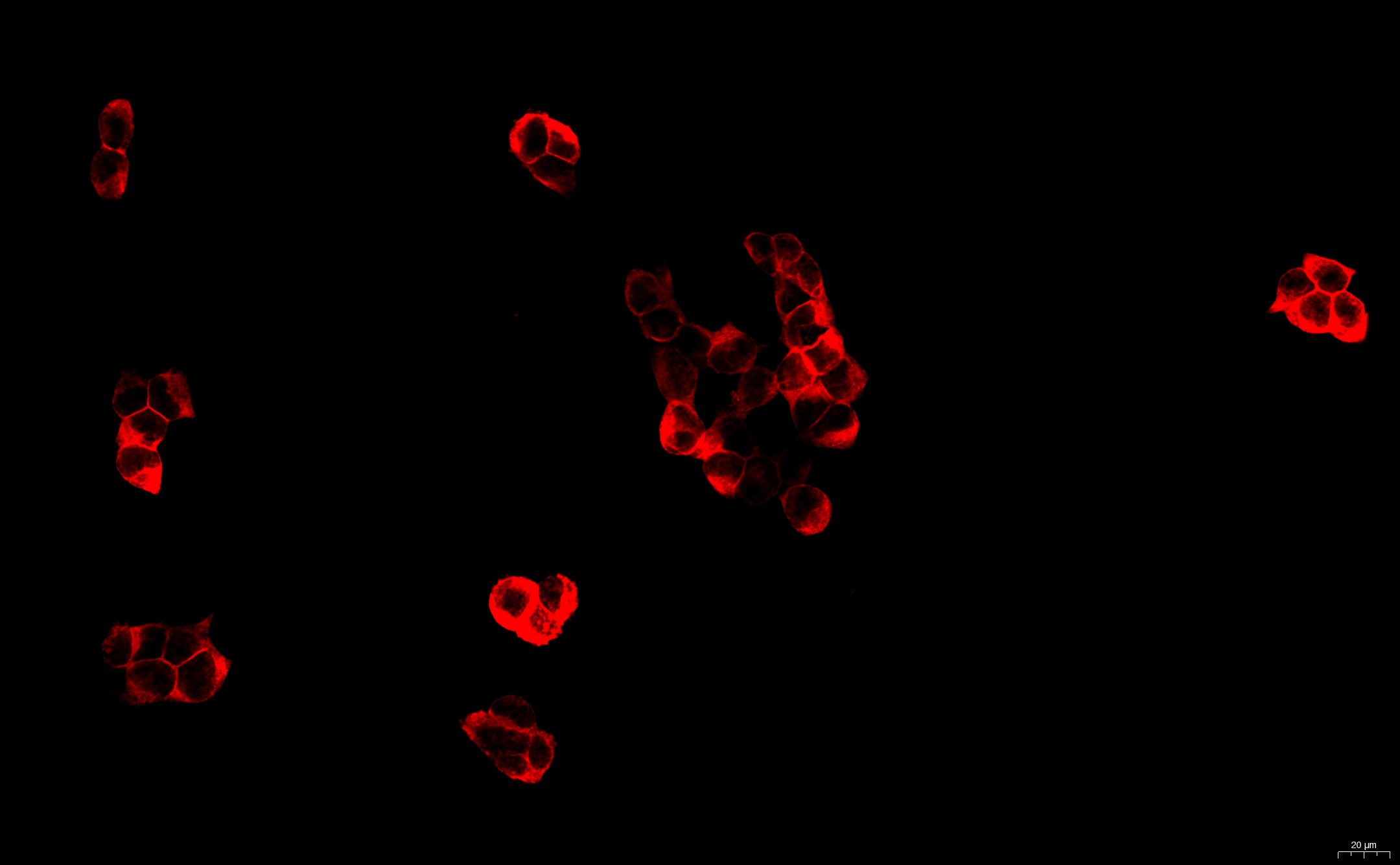

Supplement: Supplementary file 1 [file DataSheet1.zip › Supplementary/2-Immunofluorescence staining/OE group/2/1.tif]

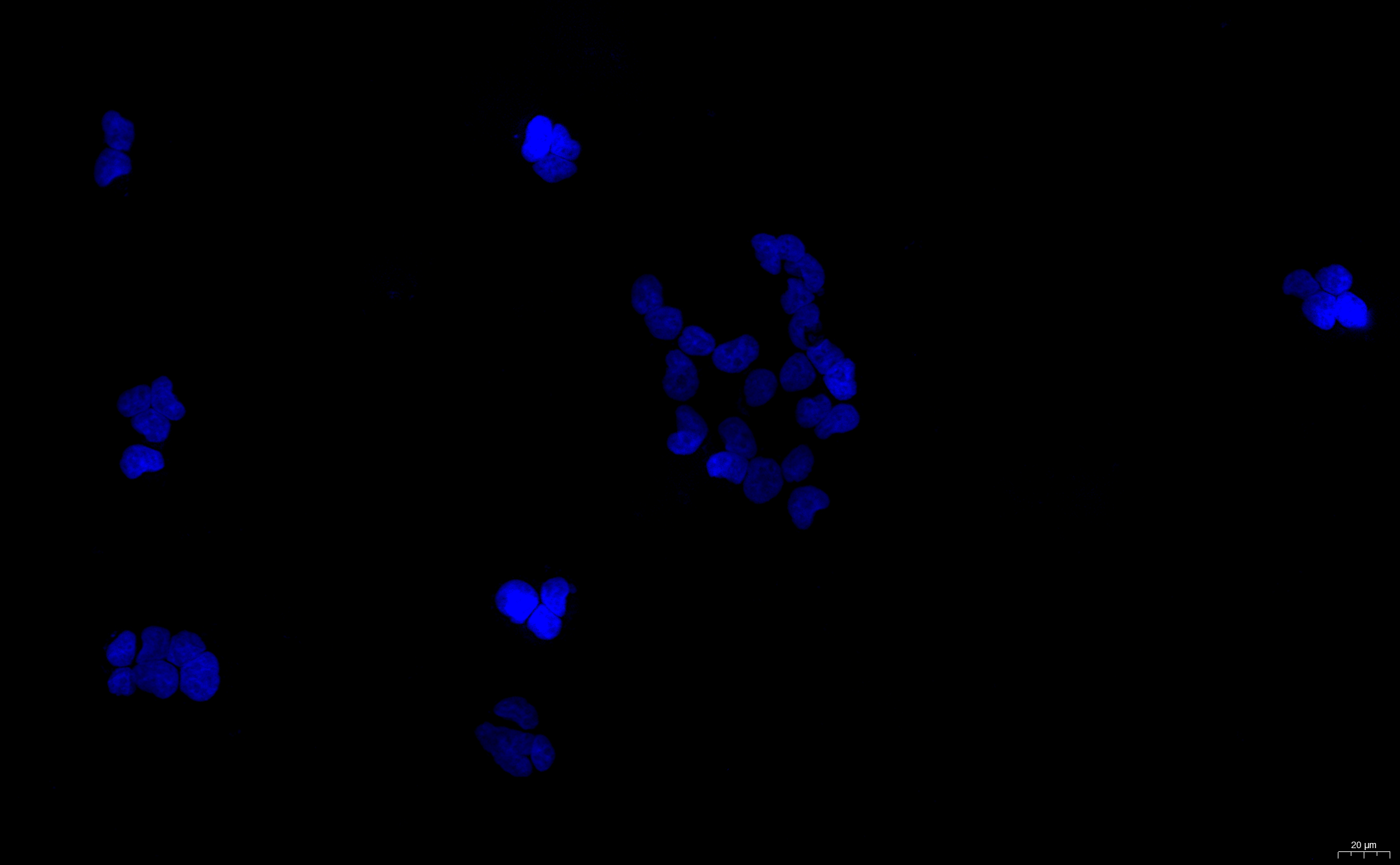

Supplement: Supplementary file 1 [file DataSheet1.zip › Supplementary/2-Immunofluorescence staining/OE group/2/2.tif]

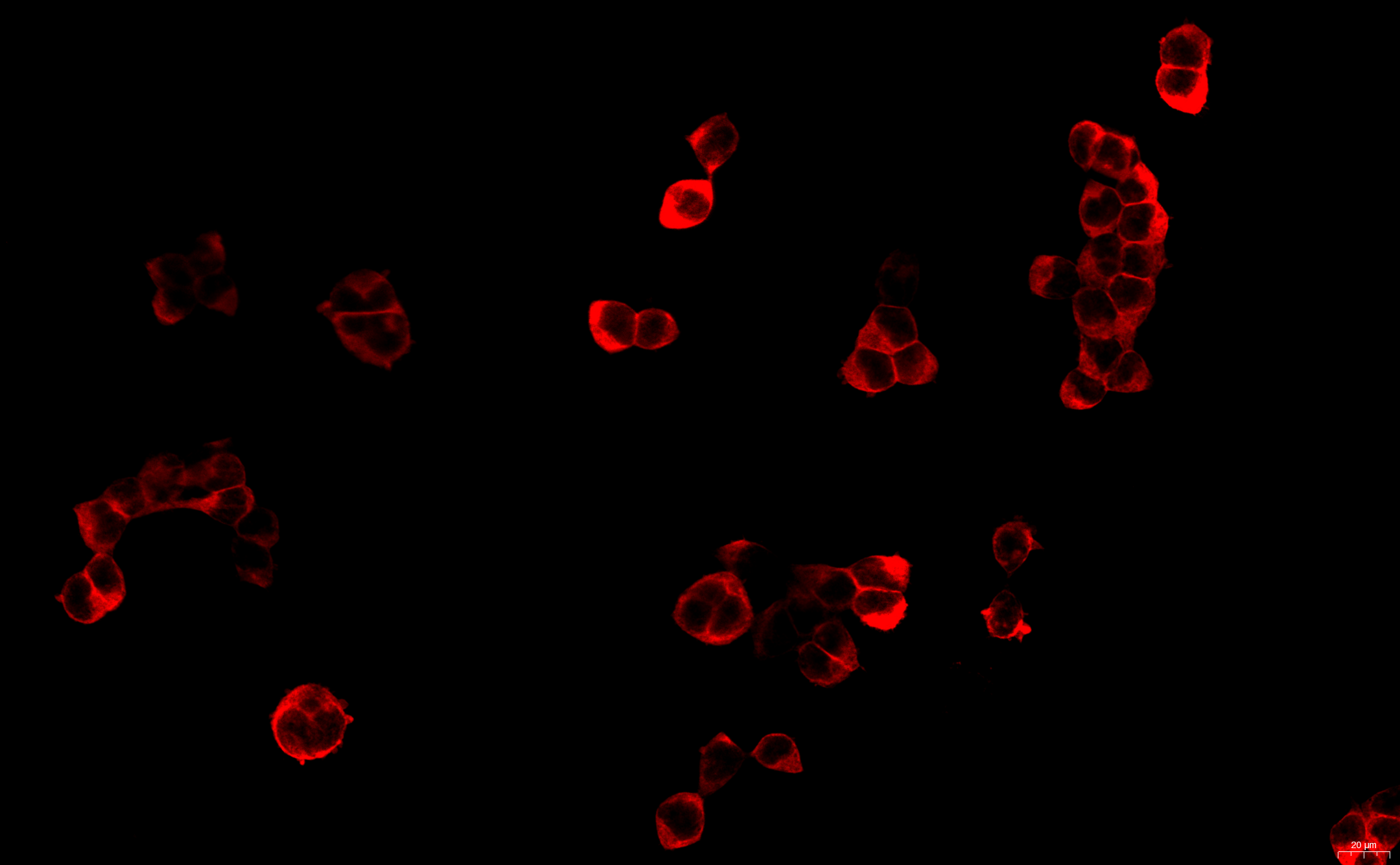

Supplement: Supplementary file 1 [file DataSheet1.zip › Supplementary/2-Immunofluorescence staining/OE group/3/1.tif]

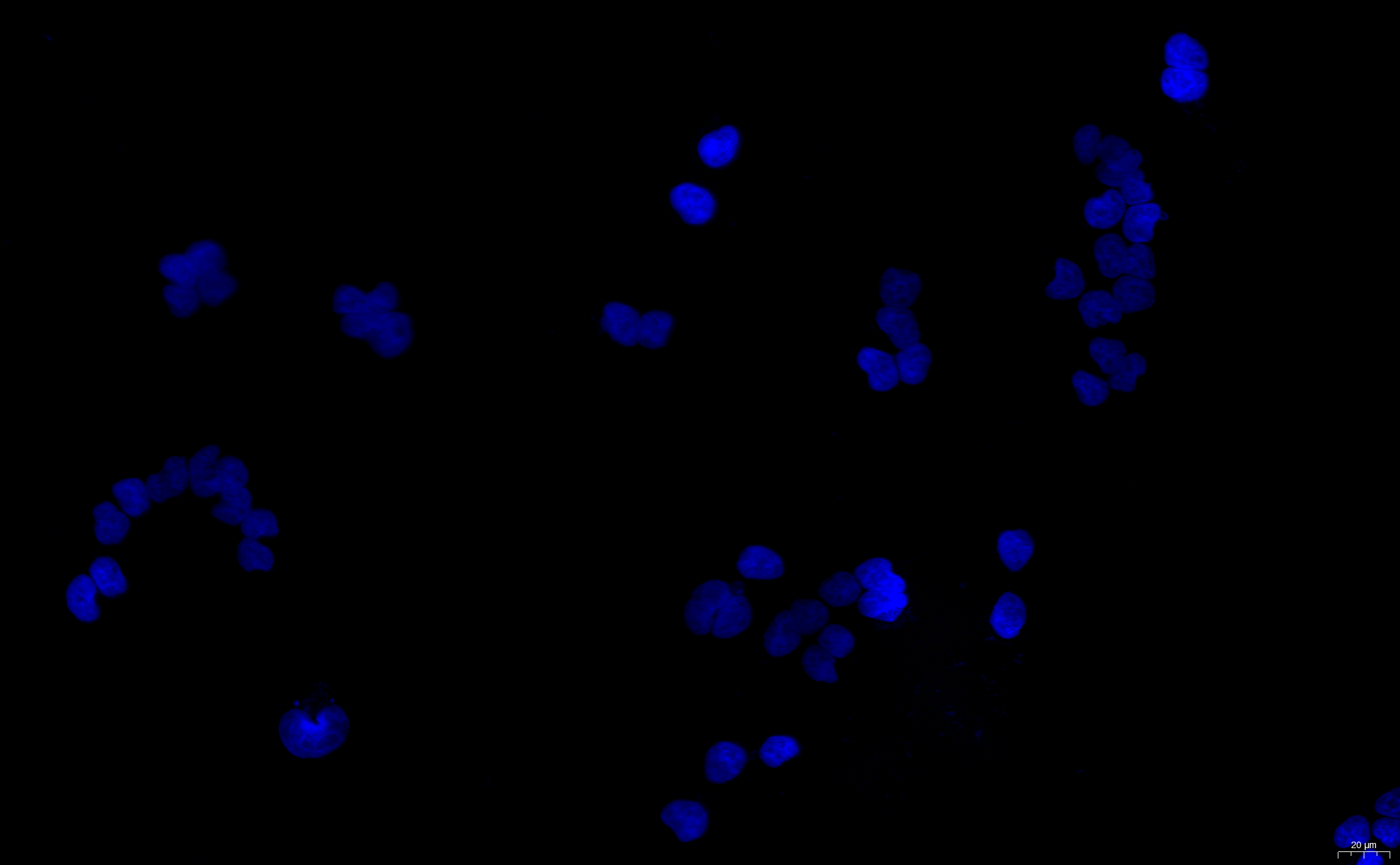

Supplement: Supplementary file 1 [file DataSheet1.zip › Supplementary/2-Immunofluorescence staining/OE group/3/2.tif]

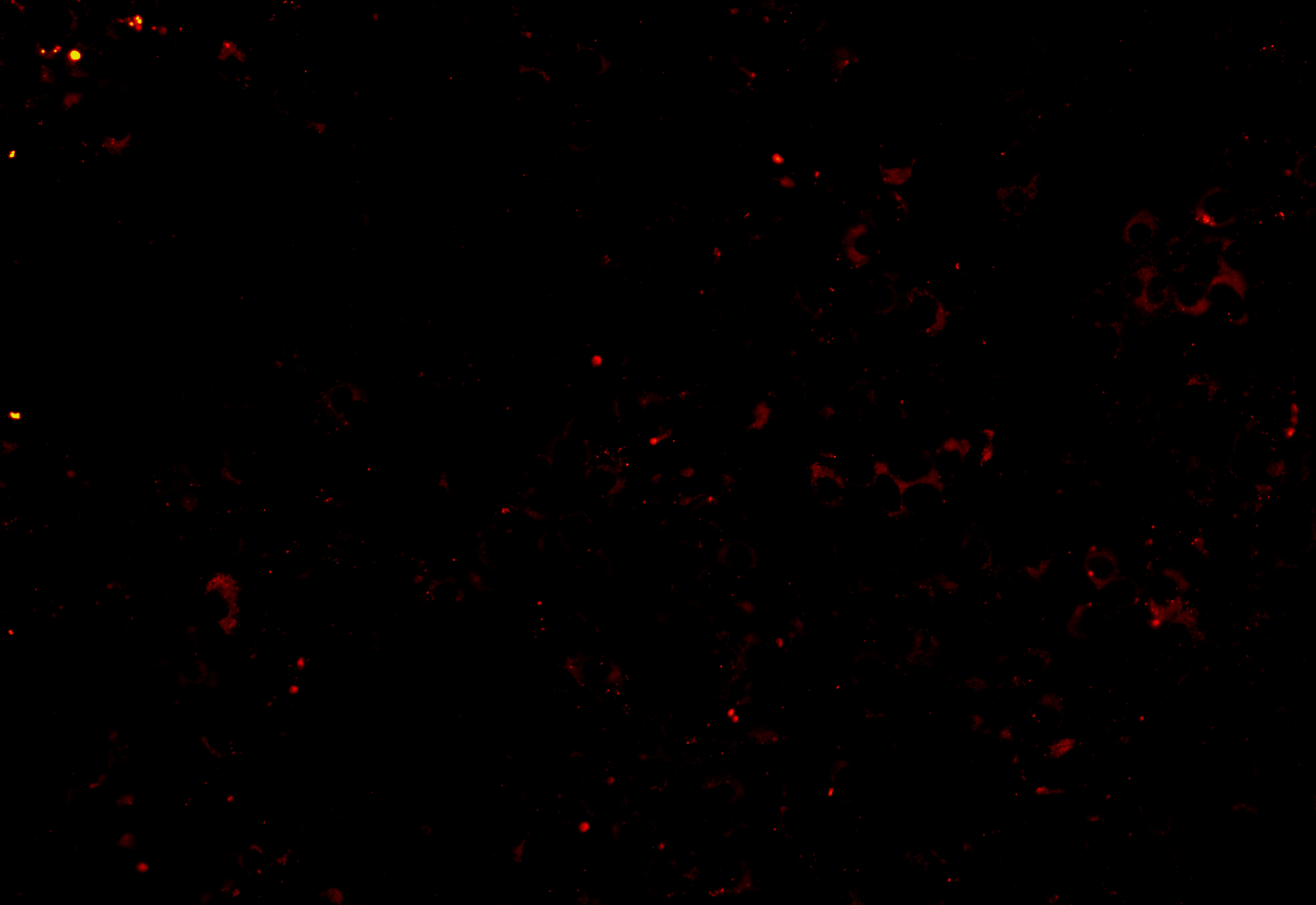

Supplement: Supplementary file 1 [file DataSheet1.zip › Supplementary/2-Immunofluorescence staining/shRNA group/1/1-1.jpg]

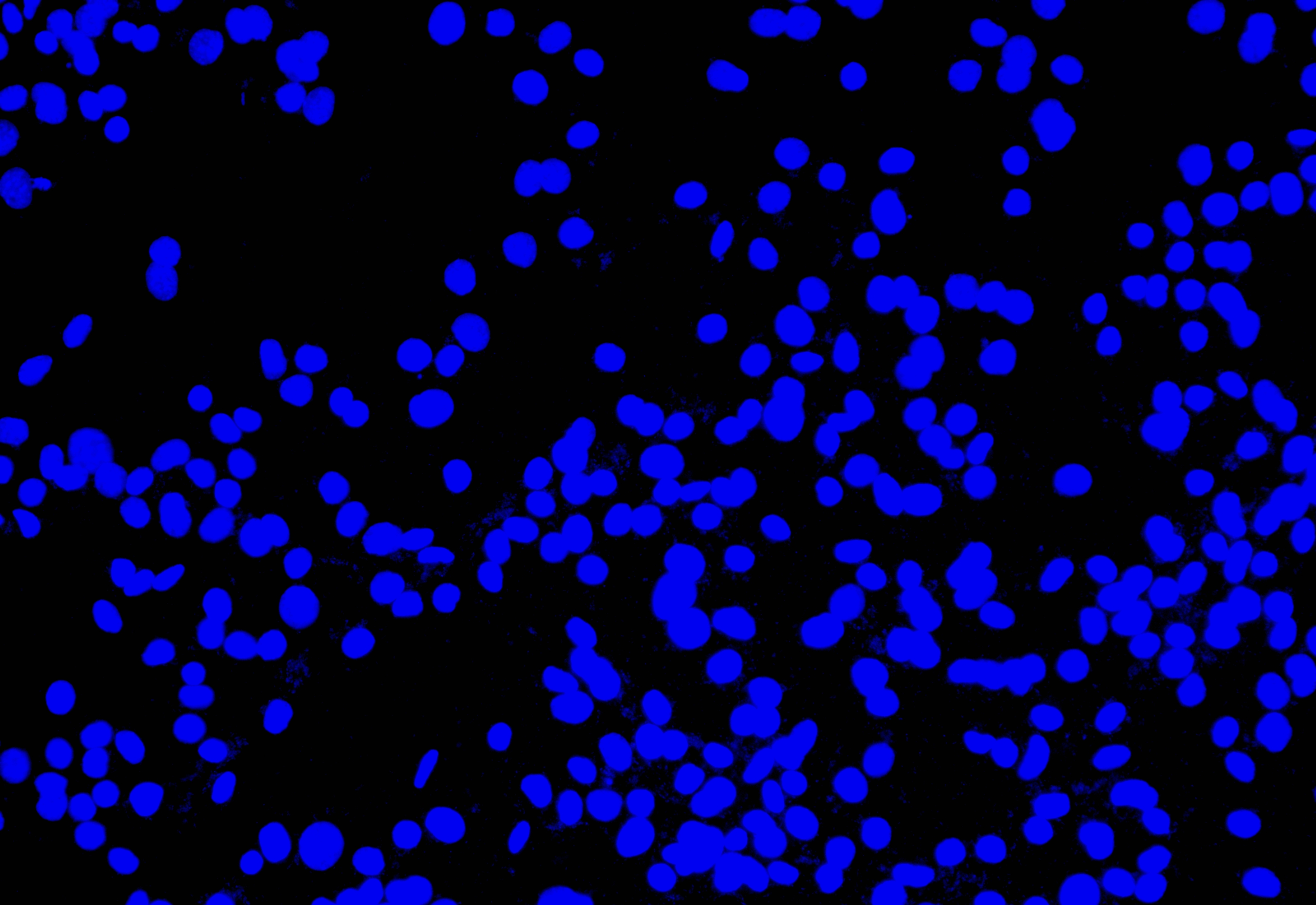

Supplement: Supplementary file 1 [file DataSheet1.zip › Supplementary/2-Immunofluorescence staining/shRNA group/1/1-2.jpg]

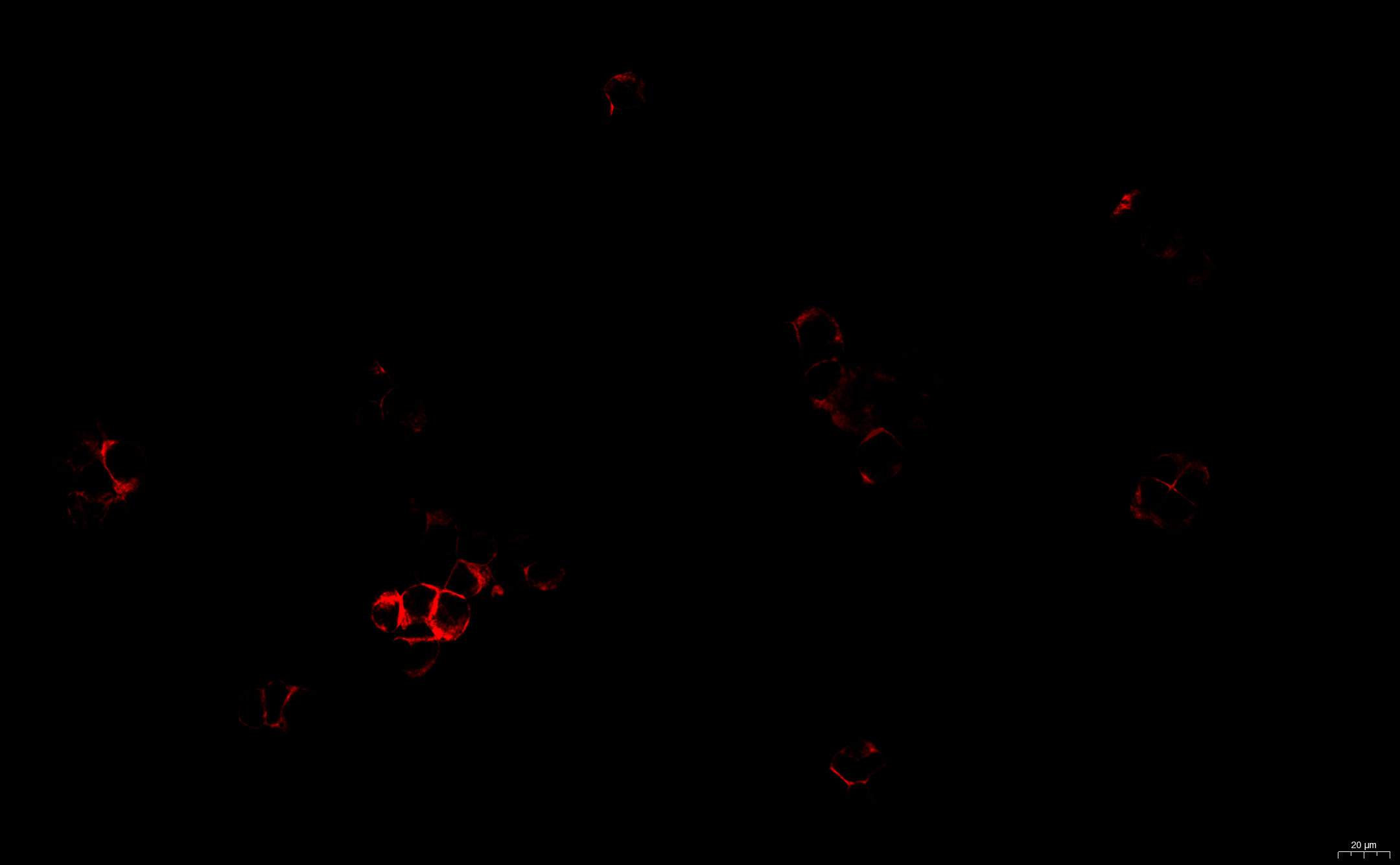

Supplement: Supplementary file 1 [file DataSheet1.zip › Supplementary/2-Immunofluorescence staining/shRNA group/2/1.tif]

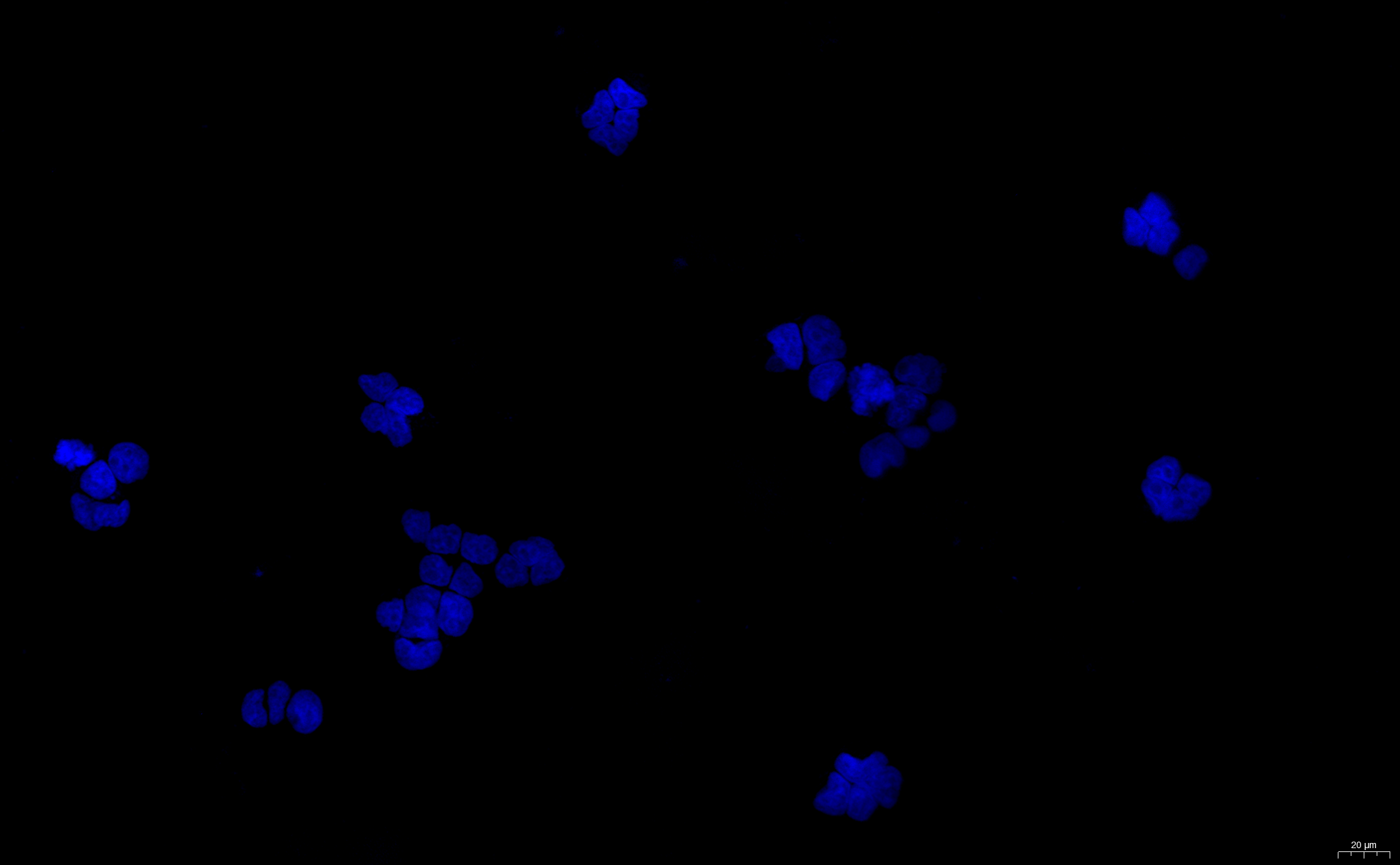

Supplement: Supplementary file 1 [file DataSheet1.zip › Supplementary/2-Immunofluorescence staining/shRNA group/2/2.tif]

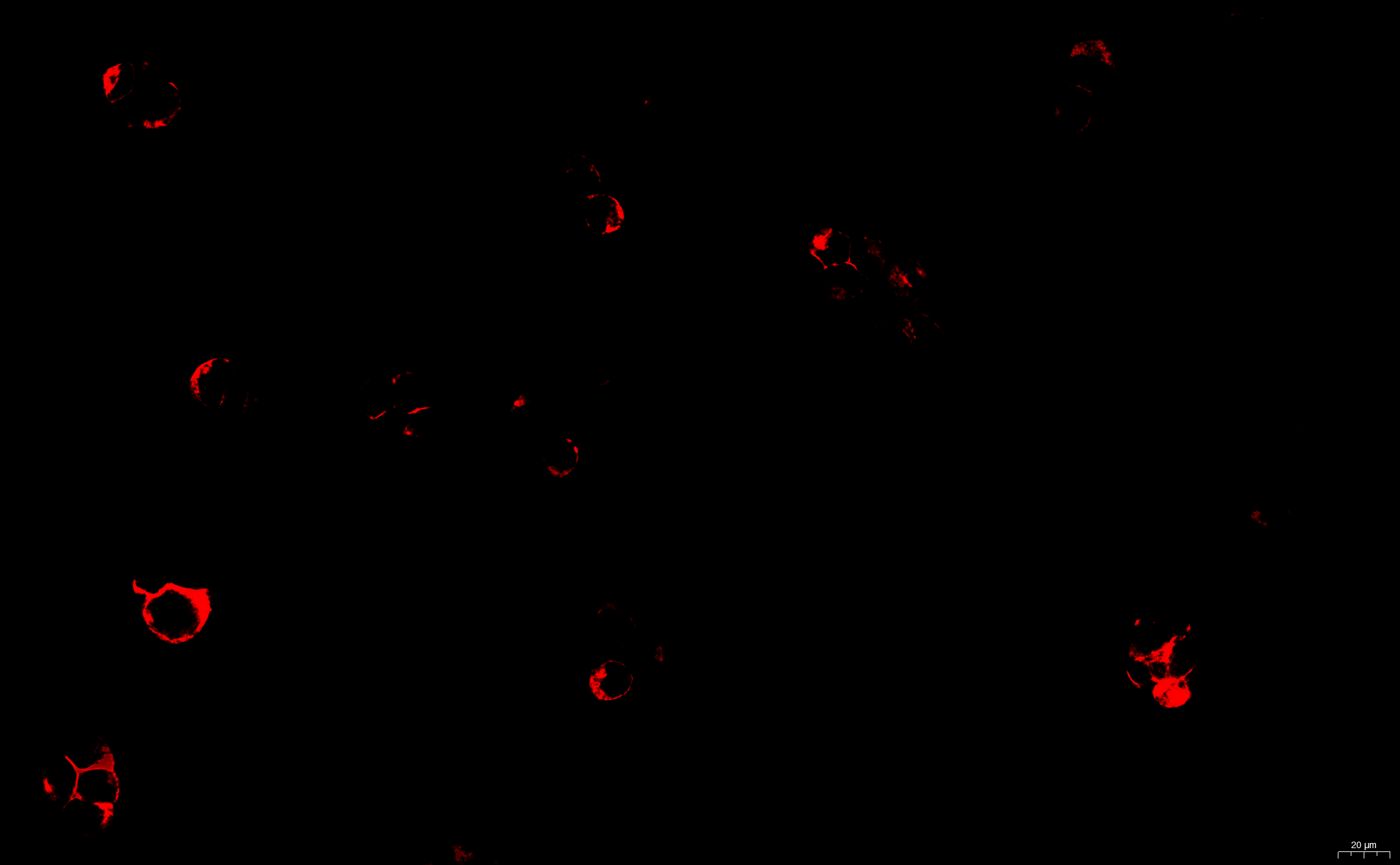

Supplement: Supplementary file 1 [file DataSheet1.zip › Supplementary/2-Immunofluorescence staining/shRNA group/3/1.tif]

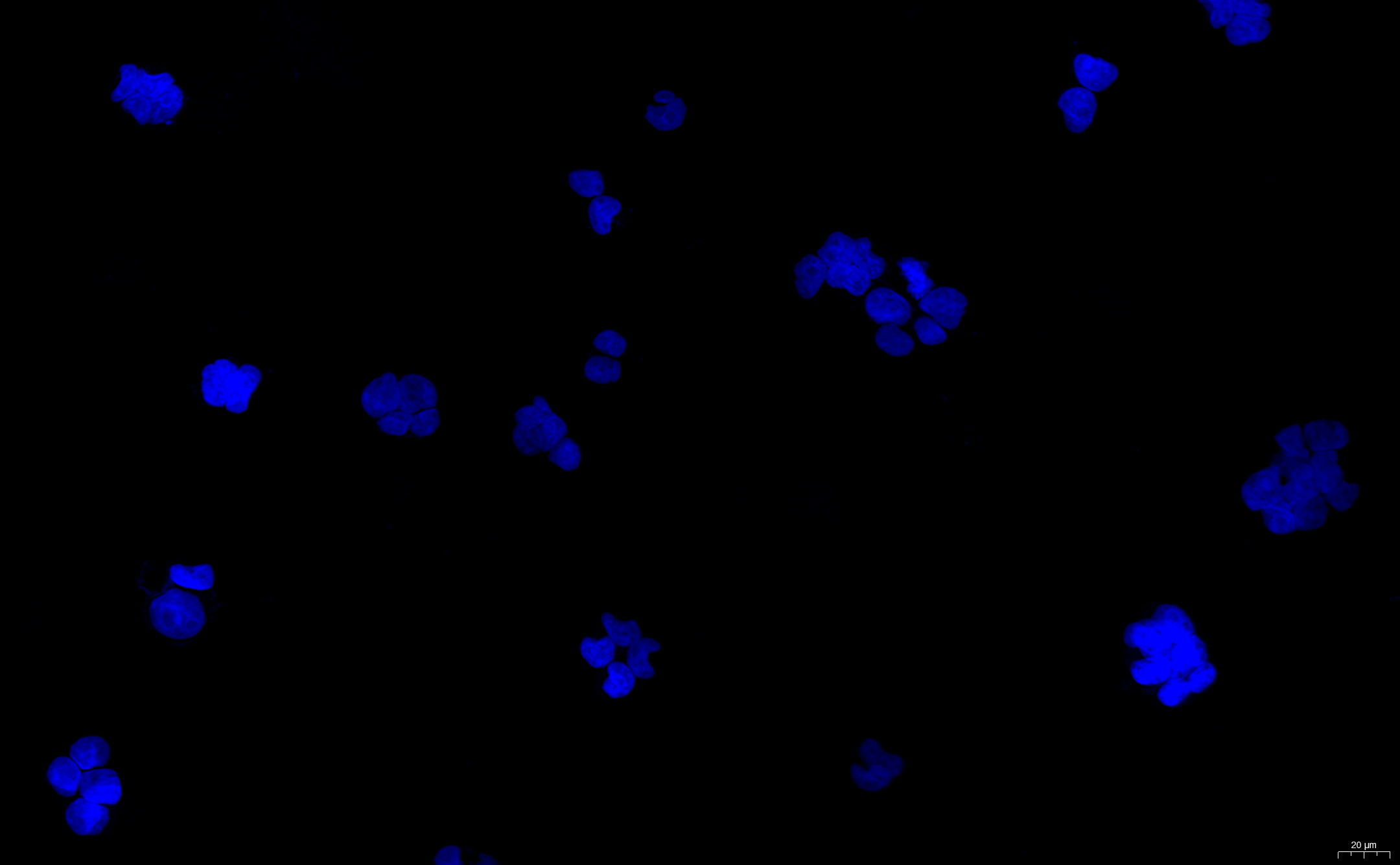

Supplement: Supplementary file 1 [file DataSheet1.zip › Supplementary/2-Immunofluorescence staining/shRNA group/3/2.tif]
